# Opposing functions of circadian protein DBP and atypical E2F family E2F8 in anti-tumor Th9 cell differentiation

**DOI:** 10.1038/s41467-022-33733-8

**Published:** 2022-10-14

**Authors:** Sang-A Park, Yun-Ji Lim, Wai Lim Ku, Dunfang Zhang, Kairong Cui, Liu-Ya Tang, Cheryl Chia, Peter Zanvit, Zuojia Chen, Wenwen Jin, Dandan Wang, Junji Xu, Ousheng Liu, Fu Wang, Alexander Cain, Nancy Guo, Hiroko Nakatsukasa, Chuan Wu, Ying E. Zhang, Keji Zhao, WanJun Chen

**Affiliations:** 1 Mucosal Immunology Section, National Institute of Dental and Craniofacial Research, National Institutes of Health, 30 Convent Drive, Bethesda, 20892 MD USA; 2 Systemic Biology Center, National Heart, Lung, and Blood Institute, National Institutes of Health, 31 Center Drive, Bethesda, 20892 MD USA; 3 Laboratory of Cellular and Molecular Biology, Center for Cancer Research, National Cancer Institute, National Institutes of Health, 37 Convent Drive, Bethesda, 20892 MD USA; 4 Experimental Immunology Branch, National Cancer Institute, National Institutes of Health, 37 Convent Drive, Bethesda, 20892 MD USA

**Keywords:** CD4-positive T cells, Tumour immunology, Gene regulation in immune cells, Interleukins

## Abstract

Interleukin-9 (IL-9)-producing CD4^+^ T helper cells (Th9) have been implicated in allergy/asthma and anti-tumor immunity, yet molecular insights on their differentiation from activated T cells, driven by IL-4 and transforming growth factor-beta (TGF-β), is still lacking. Here we show opposing functions of two transcription factors, D-binding protein (DBP) and E2F8, in controlling Th9 differentiation. Specifically, TGF-β and IL-4 signaling induces phosphorylation of the serine 213 site in the linker region of the Smad3 (pSmad3L-Ser^213^) via phosphorylated p38, which is necessary and sufficient for *Il9* gene transcription. We identify DBP and E2F8 as an activator and repressor, respectively, for *Il9* transcription by pSmad3L-Ser^213^. Notably, Th9 cells with siRNA-mediated knockdown for *Dbp* or *E2f8* promote and suppress tumor growth, respectively, in mouse tumor models. Importantly, DBP and E2F8 also exhibit opposing functions in regulating human TH9 differentiation in vitro. Thus, our data uncover a molecular mechanism of Smad3 linker region-mediated, opposing functions of DBP and E2F8 in Th9 differentiation.

## Abstract (web summary)

IL-9-producing CD4 T (Th9) cells have been implicated in allergy and tumor immunity, but how their differentiation is regulated by TGFβ is still unclear. Here the authors show that two transcription factors, DBP and E2F8, institute opposing effects on IL-9 expression downstream of TGFβ/Smad3 linker region-mediated signaling in activated mouse T cells to modulate antitumor responses.

## Introduction

Naïve CD4^+^ T cells differentiate into different T helper (Th) subsets upon TCR stimulation in the presence of different cytokines. Th9 cells are a recently identified subset of CD4^+^ T cells that produce IL-9 in response to TGF-β and IL-4 in vitro^1–3^ and have been shown to participate in the pathogenesis of allergic inflammation and asthma^4–6^ and play roles in anti-tumor immunity^7–9^. Although several transcription factors including PU.1, IRF4, Stat6, Stat5, E2A, GATA-3 and NF-κB have been reported to be involved in Th9 differentiation^10^, they are also involved in the differentiation of other Th cell subsets. It thus remains unknown as whether there are unique or master transcription factors controlling *Il9* gene transcription in Th9 cells^11–13^.

TGF-β serves an essential role in driving the development and differentiation of regulatory T cells (Tregs)^14^, Th17 cells^15^, and Th9 cells^1,3^ in the presence of IL-2, IL-6, and IL-4, respectively. Recent studies show that IL-2 selectively suppressed Th17 cell differentiation by PTEN inhibition^16^. In addition, blocking IL-6 signaling polarized TGF-β-induced Tregs with high GARP and LAP expression^17^, and IL-4 inhibits TGF-β-mediated Treg differentiation but promotes Th9 cell by HDAC9-mediated epigenetic regulation^18^. However, the underlying molecular mechanisms through which TGF-β and IL-4 signals co-operatively drive *Il9* gene transcription but not *foxp3* or *Rorγt* gene expression are not fully understood. Downstream of TGF-β signaling pathways, Smad3-dependent^9,19,20^ pathways have been reported to drive *Il9* gene expression. However, how Smad3 is activated and which part of Smad3 is required in response to TGF-β and IL-4 stimulation remains unknown. For example, Smad3 contains C-terminal (C) and linker (L) regions^21–23^, but it is unknown whether the phosphorylation of C-terminal or the linker region of Smad3 is required for *Il9* gene transcription during Th9 differentiation.

The D site of albumin promoter (albumin D-box) binding protein, also known as DBP^24–26^, belongs to the PAR bZIP (Proline and Acidic amino acid-Rich basic leucine ZIPper) family of transcription factors which includes Fos, Jun, CREB and C/EBP^26^. This transcriptional activator recognizes and binds to the sequence 5′-RTTAYGTAAY-3′ found in the promoter of genes such as albumin. Functionally, DBP is considered a circadian protein, yet it is not essential for circadian rhythm generation, but modulates important clock output genes, controlling mammalian metabolism and sleep-wake behavior. DBP has also been reported to regulate liver development and hematopoiesis. However, it is unknown whether DBP plays any role in T cell differentiation or function.

Proteins in the E2F family of transcription factors possess a wide range of functions in cell cycle regulation, cell differentiation, DNA stress response, and apoptosis. The E2F family contains typical E2Fs (E2F1-6) and atypical E2Fs (E2F7 and E2F8)^27–29^. While E2F1-6 bind to DNA preferentially as heterodimers with the related DP proteins DP1 and DP2, “atypical” E2F7 and E2F8 contain two distinct protein-binding subdomains^30^. E2F8 is considered a repressor, because it lacks the pocket protein-binding domain found in typical E2Fs. E2F8 has been reported to be required for placental development by promoting polyploidization of trophoblast giant cells^31^; It was also reported that E2F8 is a target of *microRNA-142* and is involved in controlling T cell proliferation in response to TCR stimulation^32^. However, it is unknown whether E2F8 plays a role in Th9 cell differentiation.

In this study, we discover that TGF-β and IL-4 signaling in CD4^+^ T cells induced an unexpected activation of the Smad3 linker region at the site of Serine 213 phosphorylation (pSMAD3L-Ser^213^), and that this activation is dependent on the activation of phosphorylated p38. The phosphorylation of Smad3L-Ser^213^ consequently promotes the increase in DBP expression and decrease in E2F8 expression in response to TGF-β and IL-4 signaling. Both DBP and E2F8 directly bind at the *Il9* gene promoter but played promotive and suppressive roles in *Il9* gene expression, respectively. Importantly, upon adoptive transfer, the *Dbp-*deficient and *E2f8*-deficient Th9 cells enhances and suppresses tumor growth, respectively, in mouse models of melanoma and fibrosarcoma in vivo. Notably, DBP and E2F8 also differentially regulate human Th9 differentiation in vitro, suggesting a clinical implication in cancer immunotherapy.

## Results

### Smad3 linker region phosphorylation is required for Th9 differentiation

**Fig. 1:**
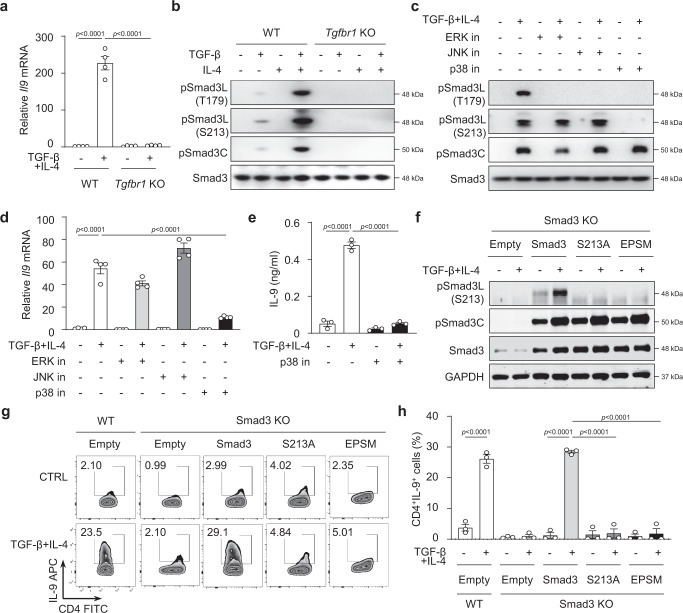
Phosphorylation of Smad3 linker Ser^213^ site is essential for Th9 differentiation by TGF-β and IL-4 stimulation. **a** Expression of *Il9* mRNA in naïve CD4^+^ T cells from *Tgfbr1*^*f/f*^ *ER*-*cre*^+^ mice that had been treated with Oil (WT) or tamoxifen (*Tgfbr1* KO) for 5 days stimulated with TGF-β plus IL-4 for 24 h. The results are presented relative to those of the control gene *Hprt*. **b** Western blot analysis of phosphorylated linker region Thr^179^ (pSmad3L-T179), Ser^213^ (pSmad3L-S213) sites and C-terminal (pSmad3C) of Smad3 and total Smad3 (Smad3) in WT and *Tgfbr1* KO CD4^+^ T cells cultured with medium, TGF-β, IL-4 or TGF-β plus IL-4 for 2 h. **c** Western blotting of Smad3 phosphorylation in normal CD4^+^ T cells pre-treated with the indicated inhibitors and then cultured with TGF-β plus IL-4 for 2 h. **d** Real time RT-PCR analysis of *Il9* mRNA in CD4^+^ T cells pre-treated with the indicated inhibitors as in **c** and then cultured with TGF-β plus IL-4 for 24 h. **e** IL-9 protein in cell culture supernatants were measured by ELISA in CD4^+^ T cells cultured with TGF-β plus IL-4 with or without p38 inhibitor for 72 h. **f** Phosphorylation of indicated Smad3 linker regions and C-terminal in Smad3^*−/−*^ CD4^+^ T cells transfected with intact WT Smad3, and S213A- or EPSM-Smad3 mutants (scheme depicted in Supplementary Fig. 2a–c), followed by TGF-β plus IL-4 stimulation for 2 h. **g** Intracellular staining of IL-9 by flow cytometry as in **f** after 72 h. **h** Summary of results in **g**. **a**, **d** These data were representative of four independent experiments or **b**, **c**, **e**–**h** pooled from three experiments. These data were analyzed by one-way ANOVA with Tukey’s test. Graphs show the mean ± SEM. Source data are provided as a Source Data file.

TGF-β signaling in the presence of IL-4 is required for the differentiation of Th9 cells^1–3,9^, as deletion of TGF-β receptor I (*Tgfbr1*^*−/−*^) in T cells abolishes *Il9* expression in naïve CD4^+^ T cells stimulated with TCR (anti-CD3 and anti-CD28) (Fig. 1a). Downstream of TGF-β receptor-mediated signaling, Smad3 has been demonstrated to play a role in Th9 differentiation as gene mutation of Smad3 (Smad3^−/−^) caused reduction of IL-9 in T cells^6,9,20^. However, the mechanism by which Smad3 activity mediates *Il9* transcription in T cells in response to TGF-β and IL-4 signaling remains unknown. Smad3 acquires its mediator activity by phosphorylation of its C-terminal (C) and/or linker (L) regions^33^ in response to different stimuli. The C-terminal Ser-Ser-X-Ser motif of Smad3 is directly phosphorylated by the activated TGF-β receptor I^34^; However, the structurally diverse linker regions of Smad3 harbor one Threonine (Thr^179^) and three Serine (Ser^204^, Ser^208^, and Ser^213^) cluster sites (Supplementary Fig. 1a), which can be phosphorylated by mitogen-induced pathways such as extracellular signal-regulated kinase (ERK), c-Jun N-terminal kinase (JNK), and p38 MAPK, in addition to TGF-β signaling^33,35^. As neither TGF-β nor IL-4 alone can stimulate T cells to transcribe the *Il9* gene, we hypothesized that the combination of TGF-β and IL-4 signals could activate the linker regions and/or C-terminal of Smad3 to induce *Il9* gene expression. To study this, we stimulated normal C57BL/6 naïve CD4^+^ T cells with anti-CD3 and anti-CD28 antibodies in the presence of TGF-β and IL-4 and examined the phosphorylation of C-terminal (pSmad3C) and linker regions Thr^179^ and Ser^213^ (pSmad3L-Thr^179^ and pSmad3L-Ser^213^). Although TGF-β treatment alone induced large amounts of pSmad3C, some degree of linker pSmad3L-Thr^179^ and minimal amounts of linker pSmad3L-Ser^213^ at 1 h, the combination of TGF-β and IL-4 resulted in reduction of pSmad3C, no alteration of pSmad3L-Thr^179^, but increase in pSmad3L-Ser^213^ compared to TGF-β treatment alone (Supplementary Fig. 1b). Surprisingly, by 2 h, the pSmad3L-Ser^213^ continued to increase and was actually the highest among the three phosphorylated sites of Smad3 in response to TGF-β and IL-4 stimulation (Fig. 1b). The total Smad3 protein was unchanged amongst all treatments (Fig. 1b and Supplementary Fig. 1b). This suggested that the combined TGF-β and IL-4 signaling optimized the activation of the linker regions especially the Ser^213^ site of the Smad3 protein. Strikingly, the phosphorylation of Smad3 C-terminal and linker regions induced by TGF-β and IL-4 was completely abolished in naïve CD4^+^ T cells deficient in TGF-β receptor I, which was accompanied by the complete failure of *Il9* gene transcription in the *Tgfbr1*^*−/−*^ T cells (Fig. 1a). The data suggest that *Il9* gene expression may require phosphorylation of the linker regions and/or C-terminal of Smad3 protein in response to TGF-β and IL-4 signaling.

We next sought to understand the downstream molecular mechanisms that mediate the phosphorylation of the C-terminal and linker regions of Smad3 in response to TGF-β and IL-4 stimulation. As it is known that the Smad3 C-terminal is directly activated with TβRI upon TGF-β stimulation (Supplementary Fig. 1b)^36^, whereas the linker regions of Smad3 can also be phosphorylated by MAPK, in addition to TGF-β signaling^33^, we hypothesized that TGF-β and IL-4 signaling could phosphorylate the linker regions of Smad3 through the aforementioned MAP kinases. For this, we firstly examined the activation of these MAPK in normal naïve CD4^+^ T cells and found that TGF-β plus IL-4 induced rapid phosphorylation of JNK, p38, and ERK; the phosphorylation peaked at 1 h, and then decreased at 2 h after stimulation (Supplementary Fig. 1c). Again, the increase in p-JNK, p-ERK and p-p38 by TGF-β plus IL-4 was completely abrogated in naïve *Tgfbr*1^*−/−*^ CD4^+^ T cells (Supplementary Fig. 1d), suggesting an important role of TGF-β signaling in the process.

We then investigated which of the activated mitogen-activated protein kinase (MAPK), i.e., p-ERK, p-JNK or p-p38, is responsible for the phosphorylation of the linker regions and C-terminal of Smad3 during Th9 differentiation. Naïve CD4^+^ T cells were stimulated with TCR and TGF-β plus IL-4 in the presence of the individual inhibitors SP600125 (inhibitor for p-JNK), U0126 (inhibitor for p-ERK), or SB203580 (inhibitor for p-p38) for 2 h, and then pSmad3C and pSmad3L-Thr^179^ or pSamd3L-Ser^213^ were determined by Western blot analysis. Inhibition of p-ERK specifically reduced pSmad3L-Thr^179^, but did not affect the pSmad3L-Ser^213^ site (Fig. 1c), consistent with the previous report^35^. Similarly, inhibition of p-JNK suppressed pSmad3L-Thr^179^ but not pSmad3L-Ser^213^ (Fig. 1c). In contrast, however, inhibition of p-p38 activity blocked the pSmad3L-Ser^213^, although it also suppressed pSmad3L-Thr^179^ (Fig. 1c). Notably, inhibition of p-ERK, p-JNK, or p-p38 failed to change pSmad3C induced by TGF-β plus IL-4 (Fig. 1c). Thus, TGF-β plus IL-4 signaling activates p-p38, which phosphorylates Smad3L-Ser^213^ and Smad3L-Thr^179^, and p-ERK and p-JNK, which phosphorylate only Smad3L-Thr^179^.

We next determined whether the phosphorylation of C-terminal or linker regions of Smad3 was required for IL-9 production in T cells. We first determined that inhibition of p-p38, which resulted in suppression of Smad3L-Ser^213^, but not inhibition of p-ERK or p-JNK, which suppressed Smad3L-Thr^179^ (Fig. 1c), significantly decreased *Il9* mRNA in CD4^+^ T cells in response to TGF-β plus IL-4 (Fig. 1d). Consistently, the IL-9 protein was also suppressed by p-p38 inhibition (Fig. 1e). The data collectively indicate that the phosphorylation of Smad3L-Ser^213^ by p-p38, but not the phosphorylation of Smad3L-Thr^179^ or of Smad3C, is required for *Il9* gene activation during Th9 cell differentiation.

To further validate the indispensable role of pSmad3L-Ser^213^ in *Il9* gene transcription, we generated Smad3 constructs that encoded serine-to-alanine mutation at the linker region Ser^213^ site (Smad3L-Ser^213A^) or encoded serine-to-alanine or threonine-to-valine mutations at all sites of the linker region T179V, S204A, S208A and S213A (EPSM). A Smad3 construct with the linker region intact was used as the wildtype control (WT) (scheme in Supplementary Fig. 2a). We transfected Smad3^−/−^CD4^+^ T cells with retrovirus carrying the WT or indicated GFP-tagged constructs to mutate the corresponding phosphorylation sites in the Smad3 linker region (Supplementary Fig. 2a–c). The CD4^+^GFP^+^ T cells were sorted and stimulated with TGF-β plus IL-4 to test the function of pSmad3L-Ser^213^ in IL-9 production. First, mutation of either Ser^213^ alone (Smad3L-Ser^213A^) or all four sites (EPSM) completely eliminated the pSmad3L-Ser^213^ in Smad3^−/−^ CD4^+^ T cells induced by TGF-β plus IL-4 (Fig. 1f), whereas the same mutations did not change any pSmad3C or the total Smad3 protein. Strikingly, mutations of Ser^213^ or EPSM almost completely eliminated IL-9 protein production (Fig. 1g, h), while the overexpression of intact WT Smad3 completely restored Th9 cells in Smad3^−/−^ T cells, confirming a required role of pSmad3L-Ser^213^ in *Il9* gene transcription. Notably, Smad3^−/−^ T cells transfected with mutations of Smad3L-Ser^213A^ or EPSM exhibited normal amounts of pSmad3C (Fig. 1f), further indicating a dispensable role of pSmad3C in *Il9* gene expression. Conversely, we overexpressed intact WT Smad3 or mutations of Smad3L-Ser^213A^ or EPSM in WT CD4^+^ T cells (Supplementary Fig. 2e–g), and showed that mutation of Ser^213^ or EPSM in the Smad3 linker regions failed to induce IL-9 production.

To confirm the dispensable function of pSmad3C, we generated Smad3 constructs without the last four amino acid residues (ΔSSVS) at the C-terminal (Supplementary Fig. 2d) and transfected them or WT Smad3 into Smad3^−/−^ T cells. WT Smad3 completely restored pSmad3C levels, but mutant ΔSSVS failed to result in phosphorylation of Smad3C (Supplementary Fig. 2h). These mutants, however, showed the exact same amounts of pSmad3L-Ser^213^ as did the WT Smad3 construct. Significantly, while the WT Smad3 construct restored *Il9* mRNA and IL-9 protein in Smad3^−/−^ T cells induced by TGF-β and IL-4, the mutants exhibited equivalent amounts of IL-9 production compared with WT Smad3-transfected T cells (Supplementary Fig. 2i, j). The data collectively eliminates a role of pSmad3C in Th9 differentiation.

In addition to IL-4, IL-6 plus TGF-β can also induce IL-9 production in CD4^+^ T cells^37^, although the amounts of IL-9 are usually lower than that in T cells stimulated with TGF-β plus IL-4 (Supplementary Fig. 1e). However, in contrast to TGF-β plus IL-4 stimulation, TGF-β plus IL-6 failed to upregulate pSmad3L-Ser^213^ (Supplementary Fig. 1f), suggesting that pSmad3L-Ser^213^ is dispensable for *Il9* gene expression mediated by TGF-β and IL-6. Stat6 has been suggested to be involved in Th9 differentiation^38^ and we noted that IL-4 plus TGF-β signaling upregulated the phosphorylation of Stat6 (p-Stat6). However, the p-p38 inhibitor failed to change the p-Stat6 induced by TGFβ and IL-4 (Supplementary Fig. 1g). In addition, under Th9-skewing conditions, Interleukin 1β (IL-1β) can activate the transcription factor Stat1 and IRF1 to promote *Il9*^39^. However, in our studies, we did not add IL-1β in our cultures, thus, Stat1 is unlikely involved in our system. Taken altogether, these results provide compelling evidence that pSmad3L-Ser^213^ is essential and sufficient for *Il9* gene expression in response to TGF-β and IL-4 signaling.

### Identification of DBP and E2F8 as critical transcription factors for *Il9* gene transcription

**Fig. 2:**
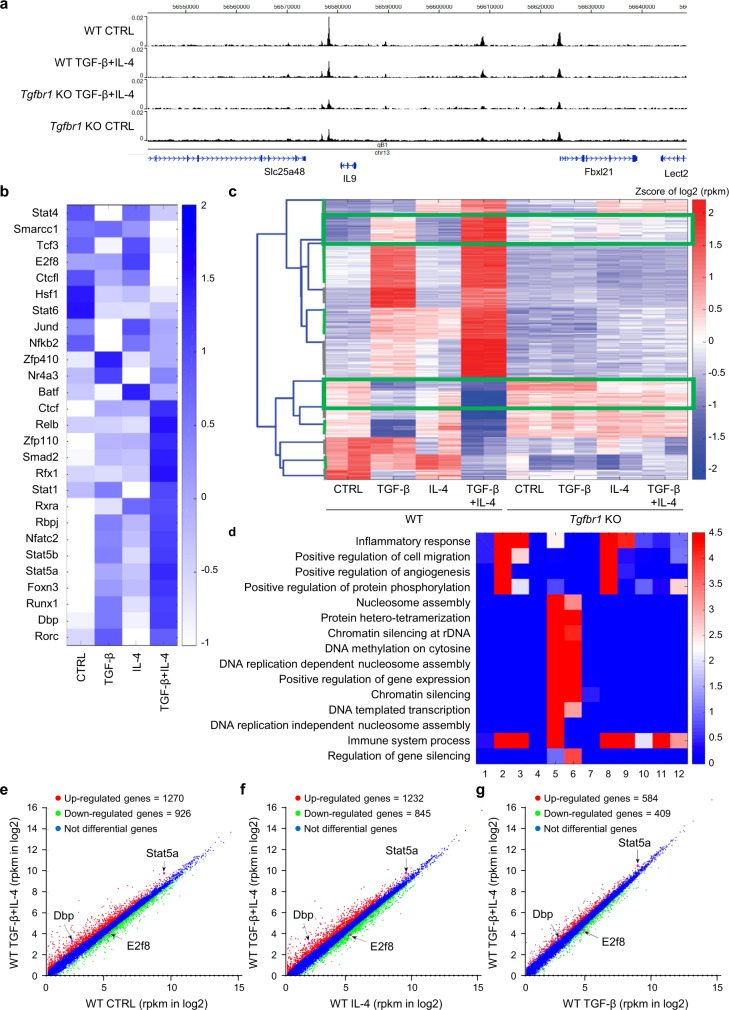
Identification of Stat5a, Dbp and E2f8 as critical transcription factors for *Il9* gene transcription. **a** A genome browser showed the genome wide profiles of DNase-seq data in four conditions including Control WT, WT TGF-β + IL-4, *Tgfbr1* KO TGF-β + IL-4 and Control *Tgfbr1* KO. **b** A heatmap showed the gene expression levels for the enriched transcription factor motifs for *Il9* in four conditions including WT Control, TGF-β, IL-4 and TGF-β + IL4. **c** A heatmap showed the clustering results (12 clusters in Supplementary Data 2) of the RNA-seq data in right conditions. The heatmap represents the Z-score of log 2-transformed rpkm expression levels. Two specific clusters were selected (with green border line) based on the distinct difference between WT TGF-β + IL-4 and all other conditions. **d** A heatmap showed the enriched GO terms for genes in each of the 12 clusters. **e** A scatter plot showed the comparison between the gene expression level of WT TGF-β + IL-4 versus control. **f** A scatter plot showed the comparison between the gene expression level of WT TGF-β + IL-4 versus WT IL-4. **g** A scatter plot showed the comparison between the gene expression level of WT TGF-β + IL-4 versus WT TGF-β.

To better understand the transcriptional regulatory elements and transcription factors that regulate the *Il9* gene expression during CD4^+^ T cell differentiation to Th9 cells in response to TGF-β and IL-4 signaling, we decided to profile the genome-wide DNase I Hypersensitive Sites (DHSs) by DNase-seq^40^ for naïve CD4^+^ T cells under the four culture conditions, namely wild-type (WT) control, WT TGF-β + IL-4, *Tgfbr1*^*−/−*^ (KO) control and KO TGF-β + IL-4. Several DHSs at the *Il9* gene locus showed decreased accessibility in the *Tgfbr1*^*−/−*^ cells (Fig. 2a). Globally, the DNase-seq analysis revealed 3208 decreased DHSs and 7023 increased DHSs in the comparison of WT TGF-β + IL-4 versus KO TGF-β + IL-4. Motif analysis of the differential DHSs by Analysis of Motif Enrichment (AME) revealed a number of enriched transcription factor (TF) motifs including those for STAT proteins, E2f8, Dbp, and JunD from gene expression (Fig. 2b). Next, we also examined the TF motifs that existed in differential DHSs around *Il9* using Find Individual Motif Occurrences (FIMO). By comparing with the previous enriched TF motifs, we found several TFs, including Stat5a, Dbp and E2f8, in WT TGF-β + IL-4 cells, suggesting that Dbp and E2f8 are potential regulators for Th9 cell differentiation.

We next measured the genome-wide gene expression profiles by RNA-seq analysis in the CD4^+^ naïve T cells under eight conditions, namely WT control, TGF-β, IL-4, and TGF-β + IL-4, and KO control, TGF-β, IL4, and TGF-β + IL-4 (Supplementary Data 1). Analysis of the RNA-seq assays revealed 12 gene clusters (Supplementary Data 2) for the eight conditions (Fig. 2c) using K-medoids clustering. Importantly, we observed that there were many more upregulated genes and down regulated genes under the WT TGF-β + IL-4 than the other conditions (Fig. 2c), indicating more extensive gene reprogramming under this condition. By performing gene ontology (GO) analysis (Gene Ontology Consortium) of the different clusters of genes, we observed that the genes in the cluster with the most increasing patterns compared to other conditions are enriched with GO terms related to immune response (Fig. 2d), suggesting the immunological roles of the Th9 cells stimulated by TGF-β plus IL-4.

Among the potential regulators found in Fig. 2b, we observed that *Dbp* was consistently increased in the WT cells stimulated with TGF-β + IL-4 compared to other culture conditions including cells stimulated with medium (WT control), IL-4 (WT IL-4), and TGF-β (WT TGF-β) (Fig. 2e–g and Supplementary Data 3). In addition, *Stat5a* was also increased in TGF-β + IL-4 treated cells (Fig. 2e–g), consistent with a previous report^41^. On the other hand, *E2f8* was consistently decreased in the WT CD4^+^ T cells cultured with TGF-β + IL-4 compared to other culture conditions (Fig. 2e–g). These results suggest that DBP and Stat5a function as activators, and E2F8 as a repressor in *Il9* gene transcription during Th9 differentiation.

**Fig. 3:**
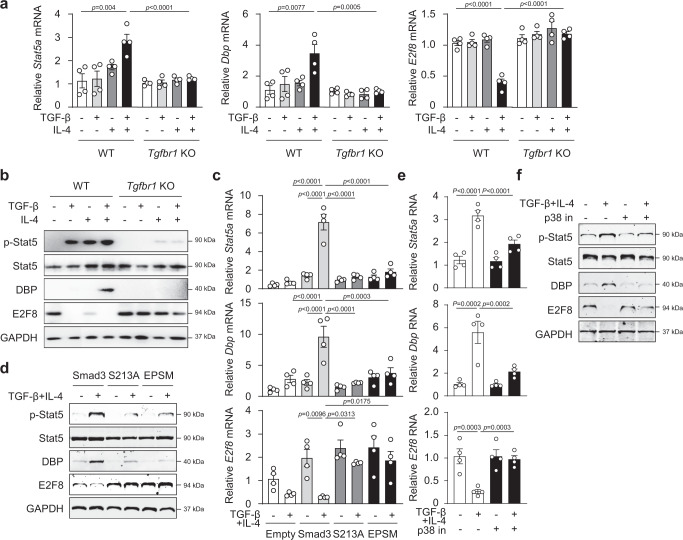
TGF-β plus IL-4 signaling increases DBP but decreases E2F8 during Th9 differentiation. **a** RT-PCR analysis and **b** Western blotting of Stat5, DBP and E2F8 in WT and *Tgfbr1* KO CD4^+^ T cells cultured with TGF-β, IL-4 or TGF-β plus IL-4. Expression of Stat5a, DBP and E2F8 during TGF-β and IL-4 stimulation in WT, S213A- or EPSM-Smad3 mutants transfected CD4^+^ T cells **c**, **d** or p38 inhibitor-pretreated CD4^+^ T cells **e**, **f** as in **a**, **b**. **a**, **c**, **e** The data were representative of four independent experiments or **b**, **d**, **f** three experiments. Data were analyzed by two-way ANOVA with Tukey’s test. Graphs show the mean ± SEM. Source data are provided as a Source Data file.

We next validated by quantitative real-time PCR that TGF-β plus IL-4, but not either one alone, significantly upregulated the *Dbp* and *Stat5a* mRNAs, but downregulated *E2f8* mRNA in CD4^+^ T cells compared to T cells treated with TCR alone with real time-PCR at 24 h (Fig. 3a). Indeed, *Stat5a*, *Dbp* and *Il9* gene expression was upregulated but *E2f8* was downregulated at 72 h after stimulation (Supplementary Fig. 3a), similar to or even better than 24 h. Importantly, these TGF-β plus IL-4-mediated changes in *Dbp* mRNAs were completely abolished in *Tgfbr1*^*−/−*^ T cells (Fig. 3a), suggesting an essential role of TGF-β signaling in the process. Consistently, TGF-β plus IL-4 enhanced the DBP proteins compared to T cells treated with TCR stimulation only (Fig. 3b), but this increase in DBP was completely abrogated in *Tgfbr1*^*−/−*^ T cells (Fig. 3b). Interestingly, pStat5 protein was upregulated by TGF-β or IL-4 as well as TGF-β plus IL-4 treatment compared to TCR-treated cells; however, the increased pStat5 was abolished in *Tgfbr1*^*−/−*^ T cells (Fig. 3b), suggesting a required role of TGF-β signaling in pStat5a activation. In marked contrast, TGF-β alone, IL-4 alone and TGF-β plus IL-4 stimulation substantially decreased E2F8 protein in WT CD4^+^ T cells compared to TCR-treated T cells (Fig. 3b); however, this change was reversed in *Tgfbr1*^−/−^ CD4^+^ T cells (Fig. 3b), suggesting a required role of TGF-β signaling in TGF-β and IL-4 mediated downregulation of E2F8. However, how TGF-β signaling also regulates IL-4-mediated E2f8 downregulation remains to be elucidated. We also compared the expression of DBP and E2F8 between Th9 and Th1, Th2, Th17, or Tregs, and found that DBP was increased in Th9 and Treg cells, and the suppression of *E2f8* mRNA and its protein was found however only in Th9 cells (Supplementary Fig. 3b, c). The data collectively indicate that TGF-β and IL-4 signaling increases DBP but decreases E2F8 during Th9 cell differentiation.

### pSmad3L-Ser^213^ regulates *Dbp* and *E2f8* expression

As the phosphorylation of Smad3L-Ser^213^ affects *Il9* gene transcription following TGF-β plus IL-4 stimulation, we next investigated the role of pSmad3L-Ser^213^ in regulating *Dbp* and *E2f8* as well as *Stat5a* expression. Firstly, analysis of the *Stat5, Dbp*, and *E2f8* promoters (from −2000 to −1 bp relative to TSS) with Genomatix software, which identifies transcription factor-binding sites showed that the promoters of these three transcription factors contained at least one Smad3-binding element, defined by the consensus nucleotide sequence GTCTAGAC, located at −1864/−1859, −1845/−1840, and −1764/−1759 (*Stat5a*); −1645/−1640 and −837/−832 (*Dbp*); −730/−725 (*E2f8*) relative to the TSS, respectively (Supplementary Fig. 4a). Chromatin immunoprecipitation (ChIP) assay showed that binding of Smad3 to the *Stat5a* and *Dbp* promoter region was significantly enriched in CD4^+^ T cells in response to TGF-β plus IL-4 (Supplementary Fig. 4b, c). Smad3 is bound at *E2f8* promoter in normal conditions, but it decreased in response to TGF-β plus IL-4 (Supplementary Fig. 4d), suggesting that TGF-β/IL-4-mediated decrease in *E2f8* expression may be due to the reduced Smad3 binding at *E2f8* gene.

Upon TGF-β and IL-4 stimulation, CD4^+^ T cells that were transfected with intact Smad3 (WT) exhibited significant upregulation of *Dbp* mRNA and DBP protein, but CD4^+^ T cells transfected with mutated Ser^213A^ or EPSM in SMAD3 linker regions completely abolished the increase in *Dbp* and DBP protein (Fig. 3c, d); On the other hand, TGF-β plus IL-4 stimulation significantly downregulated the amounts of *E2f8* mRNA and E2F8 protein in CD4^+^ T cells transfected with WT Smad3; however, this was completely reversed in the T cells transfected with mutated Ser^213A^ and EPSM (Fig. 3c, d). The increase in the expression and activation of Stat5a was also suppressed by the mutations of Ser^213A^ and EPSM (Fig. 3c, d). The data indicate that pSmad3L-Ser^213^ is the key to the upregulation of DBP expression and Stat5a activation and the downregulation of E2F8 during Th9 differentiation.

As MAPK p-p38 is the key factor in promoting pSmad3L-Ser^213^ (Fig. 1c), which plays an indispensable role in *Il9* gene transcription CD4^+^ T cells in response to TGF-β and IL-4 (Fig. 1d, e), we hypothesized that p-p38 activity affected *Stat5a, Dbp* and *E2f8* expression. We cultured naïve CD4^+^ T cells under Th9 differentiation conditions in the presence of p38-specific inhibitor and found that blockade of p-p38 activity indeed substantially suppressed the amounts of *Dbp* mRNA and DBP protein, but enhanced *E2f8* mRNA and E2F8 protein (Fig. 3e, f). Inhibition of p-p38 also decreased the *Stat5a* mRNA, and the amounts of pStat5 protein (Fig. 3e, f). The data reveal that pSmad3L-Ser^213^ is the key to the differential regulation of *Dbp* and *E2f8* in response to TGF-β and IL-4 in CD4^+^ T cells.

### Increased DBP and decreased E2F8 bindings at *Il9* gene during Th9 differentiation

**Fig. 4:**
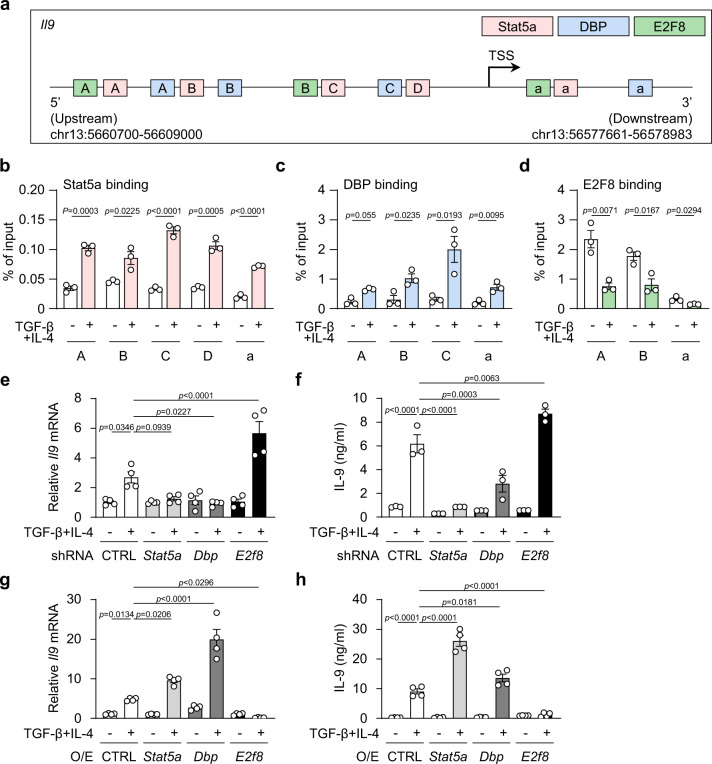
Increased DBP but decreased E2F8 binding at *Il9* gene during Th9 differentiation. **a** Identification of Stat5a, DBP and E2F8 binding elements in the *Il9* gene. **b**–**d** ChIP assay analysis for binding of Stat5a, DBP and E2F8 to the promoter region of the *Il9* during Th9 differentiation. **e**, **g** Expression of *Il9* mRNA and **f**, **h** ELISA of IL-9 and in CD4^+^ T cells transfected with *Stat5a*, *Dbp* or *E2f8* specific shRNA **e**, **f** or overexpressing virus **g, h** followed by stimulation with TGF-β plus IL-4. O/E, Overexpressed. **b**–**d**, **f** These data were representative of three independent experiments or **e**, **g**, **h** four experiments. These data were analyzed by **b**, **d** two-tailed unpaired Student’s *t* test or **e**–**h** one-way ANOVA with Tukey’s test. Graphs show the mean ± SEM. Source data are provided as a Source Data file.

In addition to assessing the impact of the changes in their expressions, we next determined that the bindings of DBP, Stat5a and E2F8 to the *Il9* gene were also affected by TGF-β and IL-4 stimulation during Th9 differentiation. Analysis of the *Il9* promoter with PROMO software (ALGGEN), which identifies transcription factor-binding sites, revealed that the *Il9* promoter region contains at least one downstream (designated as *a*) and three upstream (designated as *A, B, C)* binding sites of DBP (located at +965/+1033, −1565/−1555, −1052/−1042 and −178/−168), one downstream (designated as *a*) and two upstream (designated as *A, B*) binding sites of E2F8 (located at +43/+61, −1827/−1815 and −413/−404), and one downstream (designated as *a*) and four upstream (designated as *A, B, C, D*) binding sites of Stat5a (located at +113/+160, −1782/−1756, −1421/−1383, −362/−324 and −136/−110), relative to the transcription starting sites (TSS) (Fig. 4a). ChIP analysis showed that the bindings of all DBP and Stat5a to the *Il9* promoter region were significantly enriched in CD4^+^ T cells in response to TGF-β plus IL-4 compared with their bindings in T cells stimulated with TCR alone (Fig. 4b, c). Conversely, the binding of E2F8 to the upstream binding motifs and the downstream binding motif at the *Il9* promoter region was significantly downregulated in T cells in response to TGF-β plus IL-4 compared to its binding in cells stimulated with TCR alone (Fig. 4d). The data reveal that TGF-β plus IL-4 also enhances the direct binding of Stat5a and DBP, but decreases the binding of E2F8 at the *Il9* promoter.

### DBP promotes and E2F8 inhibits *Il9* gene expression

Next, we determined the activating function of DBP and the repressive role of E2F8 in *Il9* gene transcription during Th9 differentiation. To achieve this, we sufficiently knocked down the expression of *Dbp* and *E2f8* as well as *Stat5a* with their specific shRNAs. Strikingly, knockdown of *Dbp* with its specific shRNA resulted in a significant reduction in the amounts of *Il9* mRNA and IL-9 protein compared to control shRNA-treated T cells when stimulated with TGF-β plus IL-4 (Fig. 4e, f and Supplementary Fig. 4e). In marked contrast, knockdown of *E2f8* with its specific shRNA significantly increased *Il9* mRNA and IL-9 protein in CD4^+^ T cells compared to T cells treated with control shRNA (Fig. 4e, f and Supplementary Fig. 4e). As expected, reduction of *Stat5a* also decreased *Il9* mRNA and IL-9 protein (Fig. 4e, f and Supplementary Fig. 4e). The data indicate that DBP enhances and E2F8 suppresses *Il9* gene transcription.

To further validate the regulatory and opposing functions of DBP and E2F8 in IL-9 production, we next performed gene gain-of-function studies by overexpression of *Dbp, E2f8* and *Stat5* in T cells. The overexpression of *Dbp or Stat5* significantly enhanced Th9 cells, whereas overexpression of *E2f8* decreased Th9 cells induced by TGF-β plus IL-4. (Fig. 4g, h and Supplementary Fig. 4f). The Th9 cell differentiation was further increased at 5 days after treatment of TGF-β plus IL-4 (Supplementary Fig. 4g). Importantly, the knockdown by sh*Stat5a* or sh*Dbp* transfection in T cells significantly decreased IL-9-producing T cells, whereas sh*E2f8*-transfected T cells enhanced Th9 cells in the 5-day cultures, indicating that the transcription factors indeed affect Th9 polarization. The data collectively indicate that Stat5 and DBP acts as key enhancers and E2F8 a key repressor in *Il9* gene transcription during Th9 differentiation.

### TGF-β+IL-4 increases DBP and decreases E2F8 in CD8 Tc9 cells

In addition to Th9 cells, CD8^+^ T cells can also produce IL-9 (Tc9) in response to TGF-β and IL-4 stimulation (Supplementary Fig. 5a, b)^42^. We observed that TGF-β plus IL-4 also increased *Dbp* mRNA and DBP protein, but decreased *E2f8* mRNA and E2F8 protein expressions in CD8^+^ T cells (Supplementary Fig. 5c, d). *Stat5a* and p-Stat5a were also upregulated by TGF-β and IL-4 treatment (Supplementary Fig. 5c, d). As with Th9 cells (Fig. 1d, e), inhibition of p38 suppressed *Il9* mRNA and IL-9 protein in CD8^+^ T cells induced by TGF-β and IL-4 (Supplementary Fig. 5a, b, e, f). Importantly, inhibition of p38 also decreased the expression of Dbp and increased E2f8 in Tc9 cells respectively (Supplementary Fig. 5c, d). The expression of *Stat5a* was also suppressed by the inhibitor of p38 (Supplementary Fig. 5c, d). The data indicate a similar role of these transcription factors in IL-9 production in CD8^+^ T cells.

### DBP and E2F8 in human TH9 cell differentiation

**Fig. 5:**
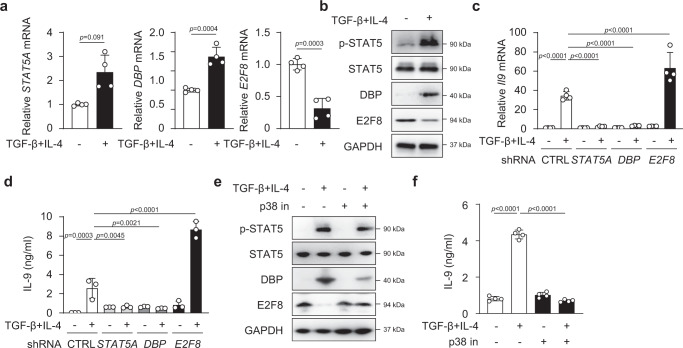
The functions of DBP and E2F8 in human TH9 differentiation. **a** RT-PCR analysis and **b** Western blotting of STAT5, DBP and E2F8 in human CD4^+^ T cells cultured with TGF-β plus IL-4 for 5 days. **c** Expression of *Il9* mRNA after 3 days and **d** ELISA of IL-9 after 5 days in human CD4^+^ T cells transfected with *STAT5A*, *DBP* or *E2F8* specific siRNA, followed by stimulation with TGF-β plus IL-4. **e** Western blotting of STAT5, DBP and E2F8 and **f** IL-9 protein were measured by ELISA in human CD4^+^ T cells cultured with TGF-β plus IL-4 with or without p38 inhibitor for 5 days. **a**, **c**, **f** These data are representative of four independent experiments or **b**, **d**, **e** three experiments. These data were analyzed by **a** two-tailed unpaired Student’s *t*-test or **c**, **d**, **f** one-way ANOVA with Tukey’s test. Graphs show the mean ± SEM. Source data are provided as a Source Data file.

We next extended our studies to investigate the expression and function of DBP and E2F8 in human TH9 cell differentiation. We isolated naïve CD4^+^ T cells from peripheral blood mononuclear cells of healthy subjects and stimulated the cells with anti-CD3 and anti-CD28 antibodies in the absence and presence of TGF-β and IL-4. As expected, and consistent with our previous findings^9^, TGF-β plus IL-4 induced *Il9* mRNA (Supplementary Fig. 6a) and IL-9 protein (Supplementary Fig. 6b, c) in human T cells. Similar to murine CD4^+^ T cells, TGF-β plus IL-4 increased the mRNAs of *DBP* and *STAT5A*, but decreased *E2F8* mRNA (Fig. 5a). Western blot analysis revealed a substantial upregulation of DBP and pSTAT5 proteins, and downregulation of E2F8 protein in human CD4^+^ T cells stimulated with TGF-β plus IL-4 compared to cells stimulated with TCR alone (Fig. 5b). Importantly, knockdown of *DBP* and *E2F8* genes in human T cells also suppressed and enhanced *Il9* gene expression and IL-9 production during TH9 differentiation, respectively (Fig. 5c, d). As expected, knockdown STAT5A also suppressed TH9 cells (Fig. 5c, d). Consistent with mouse CD4^+^ T cells, blockade of p-p38 with a specific inhibitor also decreased amounts of pSTAT5 and DBP, but increased E2F8 protein (Fig. 5e), and consequently resulted in downregulation of IL-9 production in human CD4^+^ T cells treated with TGF-β and IL-4 (Fig. 5f). The data indicate that DBP and E2F8 are also required to enhance and repress *Il9* gene expression in human T cells, respectively.

### Opposing functions of DBP and E2F8 in Th9-mediated anti-tumor response in mice

**Fig. 6:**
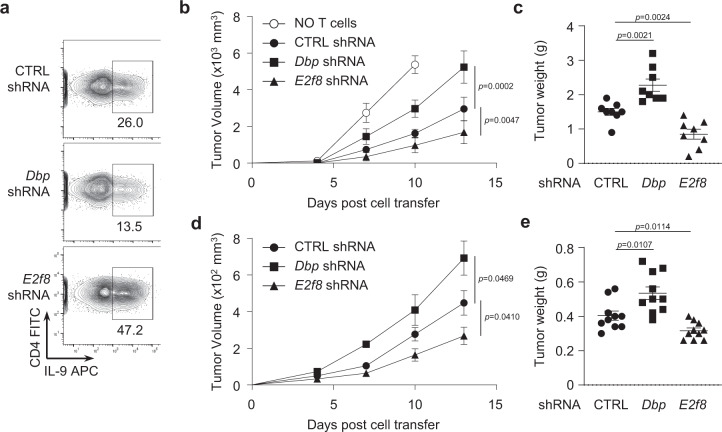
DBP and E2F8 play opposite roles of Th9 cell mediated anti-tumor activity in vivo. **a** Intracellular staining of IL-9 produced by CD4^+^ T cells with indicated shRNA transfection before cells were transferred into *Rag1*^*−/−*^ mice. Tumor growth over time in 8-week-old male and female *Rag1*^*−/−*^ mice given intravenous transfer of CTRL, *Dbp* or *E2f8* shRNA treated CD4^+^ T cells along with simultaneous subcutaneous injection of **b** B16 melanoma cells (male: *n* = 5 and female: *n* = 3 mice per group) or **d** MCA205 fibrosarcoma cells (male: *n* = 5 and female: *n* = 5 mice per group) on day 0. **c**, **e** Tumor weight at the end of experiments from **b** and **d**. These data were analyzed by two-tailed unpaired Student’s *t*-test. These data were representative of two independent experiments. Graphs show the mean±SEM. Source data are provided as a Source Data file.

Having established opposing functions for DBP and E2F8 in Th9 cell differentiation in vitro, we next determined the roles of DBP and E2F8 in Th9 cell-mediated anti-tumor activity in vivo. As Th9 cells have been well documented to suppress tumor growth^8,9,43^, we firstly utilized an experimental mouse model of melanoma in which *Rag1*^−/−^ mice were injected with B16F10 melanoma cells and treated with adoptive transfer of Th9 cells^9^. Naïve CD4^+^ T cells were genetically engineered to knockdown *Dbp* or *E2f8* genes by retroviral transfection of their specific shRNAs; a control shRNA was used as a control of transfection. These genetically engineered T cells were subsequently stimulated with TGF-β and IL-4 to induce Th9 cells (Fig. 6a). Consistent with what we showed earlier (Fig. 4), we found that knockdown of *Dbp* decreased, and reduction of *E2f8* increased, Th9 cell differentiation (Fig. 6a). We then adoptively transferred these engineered T cells into *Rag1*^–/–^ recipient mice by intravenous injection, and then subcutaneously injected B16 melanoma cells into the same mice. Consistent with our previous report^9^, mice without transfer of T cells consistently showed aggressive tumor growth (Fig. 6b), whereas treatment of mice with T cells transfected with the control shRNA, resulting in normal Th9 cells, significantly decrease tumor growth (Fig. 6b). Strikingly, however, mice that received *Dbp* shRNA-engineered T cells that decreased IL-9 (Fig. 6a) were less effective at eliminating tumor than normal Th9 cells (Fig. 6b), whereas mice that received *E2f8* shRNA-engineered T cells that increased IL-9 (Fig. 6a) showed significantly slower tumor growth, when compared to mice that received T cells treated with the control shRNA (Fig. 6b). Consistent with the growth curve, analysis of the tumor weight at the end of the experiment showed that treatment of tumor-bearing mice with T cells deficient in *Dbp* significantly increased tumor weight, but treatment of tumor-bearing mice with T cells deficient in *E2f8* profoundly decreased tumor weight (Fig. 6c). Analysis of T cells isolated from the tumor tissues as well as from draining lymph nodes and spleens revealed no changes in the frequencies of IFN-γ- and TNFα-producing Th1 cells, IL-4-producing Th2 cells, IL-17-producing Th17 cells or Foxp3^+^ Tregs among all the groups of mice (Supplementary Fig. 7a–e), suggesting that the antitumor effects were primarily due to the injected Th9 cells, but not other T cell subsets.

The same anti-tumoral and pro-tumoral effects of DBP and E2F8 on Th9 cells were verified in another experimental model of MCA205 fibrosarcoma. Mice bearing the fibrosarcoma and receiving *Dbp* shRNA-engineered T cells showed less effective at eliminating tumor than normal Th9 cell-injected mice (Fig. 6d) and larger tumor size and increased weight (Fig. 6e) than did mice that received control shRNA-treated T cells, whereas mice that received *E2f8* shRNA-engineered T cells showed significantly slower tumor growth (Fig. 6d) and reduced weight **(**Fig. 6e**)** than did mice that received control shRNA-treated T cells. Analysis of the intratumor T cells isolated from all the mice showed similar numbers of Th1, Th17 and Tregs (Supplementary Fig. 7f–j) as was seen in the melanoma model. Intriguingly, however, we noticed that the intratumor T cells from mice treated with *Dbp*-knockdown Th9 cells exhibited a slight but statistically significant decrease in IL-4 production, but the intratumor T cells from mice treated with *E2f8*-knockdown Th9 cells showed increased IL-4 secretion compared to the mice treated with control T cells (Supplementary Fig. 7h) in this fibrosarcoma model, although the mechanisms and functional significance of this observation remains unknown.

As seen in vitro, mice that received *Stat5a* shRNA-engineered T cells also exhibited significantly faster tumor growth (Supplementary Fig. 8a, b), suggesting that Stat5 indeed also plays an important role in Th9-mediated antitumor activity. We also investigated whether IL-9 affects anti-tumor therapeutic effects in vivo in the MCA205 fibrosarcoma model and we found indeed that neutralization of IL-9 with anti-IL-9 antibody abolished the anti-tumor therapeutic effects of *E2f8*-knockdown Th9 cells (Supplementary Fig. 8c, d). Importantly, knockdown of *Dbp* and *E2f8* genes in CD4^+^ T cell suppresses and promotes *Granzyme b*, respectively, in Th9 cells in vitro (Supplementary Fig. 8e) and also in Granzyme B^+^ T cells in tumor-bearing mice in vivo (Supplementary Fig. 8f). The data collectively reveal the opposing activities between DBP and E2F8 in the regulation of Th9 cells in cancer immunotherapy in vivo.

## Discussion

Th9 cells as a recently identified subset of CD4^+^ T effector cells are involved in the pathogenesis of allergy and asthma and play an important role in anti-tumor immunity^1,11^. In this paper, we have discovered transcription factors DBP and E2F8 to be the key activator and repressor, respectively, for *Il9* gene expression, and these transcription factors are controlled by phosphorylated Smad3 linker region Ser^213^ (pSmad3L-Ser^213^) downstream of TGF-β and IL-4 signaling during Th9 differentiation.

Several unprecedented conclusions can be drawn from these findings. Firstly, the phosphorylation and activation of Smad3L-Ser^213^ rather than the Smad3 C-terminal is required for *Il9* gene expression in Th9 cells. Supporting this conclusion are several lines of experimental evidence. At early time point (1 h), only pSmad3L-Ser^213^, but not pSmad3C and pSmad3L-Thr^179^, is substantially upregulated by TGF-β plus IL-4 treatment compared to TGF-β alone; pSmad3C is actually decreased in TGF-β plus IL-4 treated T cells compared to TGF-β-treated cells. By 2 h, the pSmad3L-Ser^213^ continues to increase and is the highest among the three phosphorylated sites of Smad3 in response to TGF-β and IL-4. Although both the linker region (sites Ser^213^ and Thr^179^) and the C-terminal of Smad3 can be phosphorylated by TGF-β plus IL-4 signaling, only the suppression of pSmad3L-Ser^213^ by either p38 inhibitor or mutation of Ser^213A^, both which do not affect pSmad3C levels, blocks the induction of *Il9* gene expression in CD4^+^ T cells. Additionally, the phosphorylation of other sites of Smad3 linker region such as Smad3L-Thr^179^, is not required for *Il9* gene expression, as blocking the phosphorylation of Smad3L-Thr^179^ with ERK- and JNK- specific inhibitors that do not change the pSmad3L-Ser^213^ and fail to change Th9 cell generation. The key role of pSmad3L-Ser^213^ in *Il9* gene induction is further confirmed by the fact that mutation of Ser^213A^ causes almost complete abrogation of *Il9* gene induction as seen by the mutation of all the sites of the linker region (EPSM) of Smad3. Furthermore, the fact that pSmad3L-Ser^213^ is indispensable to controlling the expression of DBP and E2F8, which directly bind to the *Il*9 gene and respectively activates and represses *Il9* gene transcription, provides unequivocal evidence to establish pSmad3L-Ser^213^ as the key factor linking the signaling from TGF-β and IL-4 to Th9 generation. Finally, a role of pSmad3C in Th9 cell differentiation is excluded by the fact that mutation of the Smad3 C-terminal to abolish the phosphorylation of Smad3C fails to change *Il9* gene expression in CD4^+^ T cells in response to TGF-β and IL-4. The discovery of the key role of pSmad3L-Ser^213^ rather than pSmad3C in *Il9* gene expression is unexpected, as all of the published studies thus far have only focused on the activation of the C-terminal of Smad3/2 (pSmad3C and/or pSmad2C), which can be activated by TGF-β and IL-4 signaling, and pSmad3C is assumed to be evidence of Smad3 involvement in Th9 cell differentiation. Thus, there have been no publications addressing the role of the Smad3 linker region in Th9 cell differentiation. This finding is also significant since this is the first evidence that the Smad3 linker region but not pSmad3C is essential and sufficient in Th9 cell differentiation. This finding is consistent with the observation that pSmad3C can be directly activated by TGF-β signaling through TGF-β receptor I, which is critical for the generation of Foxp3^+^ Tregs and RORγt^+^ Th17 cells^44,45^, but the linker region of Smad3 can also be activated by mitogen-induced pathways including inflammatory cytokines and factors^21,23,46^ in addition to TGF-β signaling, as exemplified here by IL-4 and TGF-β. Of note, the requirement for pSmad3L-Ser^213^ seems to be specific to IL-9 production induced by IL-4 plus TGF-β signaling, as IL-6 plus TGF-β signaling, which also induces some IL-9 in T cells^37^, does not require pSmad3L-Ser^213^ participation. These findings pave the way for elucidating the exact molecular mechanisms related to the involvement of the Smad3 linker region in the differentiation of other T cell subsets.

Secondly, MAPK p38 is the key to phosphorylating and activating pSmad3L-Ser^213^ in mediating the signaling downstream of TGF-β and IL-4 stimulation. In the search for the upstream activators that incite the phosphorylation of the Smad3 linker region, we focus on the ERK, JNK,and p38, which have been well documented to be able to regulate the phosphorylation of the Smad3 linker region in response to different stimuli in the presence and absence of TGF-β signaling^21,23,46^. For example, it has been reported that EGF induces Smad3L phosphorylation at Thr^179^, Ser^204^ and Ser^208^ through ERK^47^, while cyclin-dependent kinase (CDK) can phosphorylate Smad3 at all four linker sites, including Ser^213^, in epithelial cells^48^. Although TGF-β also induces phosphorylation on Smad3L-Thr^179^, Ser^204^, Ser^208^, but not Ser^213^, CDK instead of ERK activity is required for TGF-β-induced Smad3L phosphorylation^35^. In this study, we have shown in CD4^+^ T cells that the combination of TGF-β and IL-4, but neither one alone, induces a fast and optimal phosphorylation of JNK, ERK and p38 that appears as early as 15-30 min and peaks at 1 h after stimulation. However, despite the phosphorylation of all three MAPKs, only blocking of p-p38, but not p-ERK and p-JNK, with their specific inhibitors suppresses *Il9* gene expression induced by TGF-β and IL-4. The reason for this is that inhibition of p-ERK and p-JNK only suppresses the phosphorylation of Smad3L-Thr^179^, but not Smad3L-Ser^213^, thus eliminating them as the key factors in *Il9* gene expression. In contrast, blockade of p38 phosphorylation with its specific inhibitor suppresses the pSmad3L-Ser^213^, although it also suppresses pSmad3L-Thr^179^. Thus, the findings identify the key role of p38 in promoting *Il9* gene transcription through pSmad3L-Ser^213^ in Th9 differentiation. Of note, we have also excluded a major role of Stat6 in the cascade of p-p38/Smad3L-Ser^213^ in *Il9* gene expression, because blockade of p-p38 does not significantly change the amounts of pStat6 in T cells. This finding, however, does not preclude a role for Stat6 in IL-9 production through other pathways^1,38,41^.

Significantly, circadian DBP activates, and E2F family member E2F8 represses, *Il9* gene transcription during Th9 differentiation. Recently, it was reported that DBP might be involved in mucosal ILC3-derived IL-22 production, although the underlying molecular mechanisms remain unclear^49,50^. By using non-biased and global DNA-seq and RNA-seq analyses in CD4^+^ T cells stimulated with TGF-β and IL-4 in comparison to normal T cells treated with TCR stimulation alone and to *Tgfbr1*^−/−^ CD4^+^ T cells, we have identified that *Dbp* is upregulated and *E2f8* downregulated in normal CD4^+^ T cells during Th9 cell differentiation. In addition, Stat5a is also increased in Th9 cells consistent with a previous report^41^, so we used Stat5 as a positive control in our paper. In addition, Stat5 activation is also involved in promoting *Foxp3* gene expression in response to TGF-β and IL-2^51–53^, thus Stat5 is not the specific inducer for Th9 differentiation. The increase in *Dbp* and decrease in *E2f8* mRNA induced by TGF-β and IL-4 have been verified by real-time quantitative PCR. At the protein level, DBP is substantially increased and E2F8 decreased in T cells stimulated with TGF-β and IL-4. Strikingly, the changes in DBP and E2F8 are absolutely dependent on TGF-β signaling, as *Tgfbr1*^−/−^ T cells exhibit complete failure of DBP upregulation and E2F8 downregulation in response to TGF-β plus IL-4 signaling. This TGF-β plus IL-4 signaling-mediated increase in DBP and decrease in E2F8 also occurs during IL-9 production in Tc9, suggesting a general function of these two transcription factors in *Il9* gene expression. We found that Th9 cells exhibited highest DBP, although Tregs also showed some increase in DBP. Interestingly and importantly, the suppression of E2F8 was found only in Th9 cells, suggesting it is more specific in Th9 cell differentiation. Further mechanistic studies revealed that the changes between DBP and E2F8 were critically mediated by the p-p38-pSmad3L-Ser^213^ axis, as blockade of p-p38 activity or mutation of Smad3L-Ser^213A^ abolished the upregulation of DBP and downregulation of E2F8 in T cells induced by TGF-β and IL-4. The findings that Smad3 directly binds to the promotors of *Dbp* and *Stat5a* genes as determined by ChIP provides evidence of direct interaction between pSmad3L-Ser^213^ and the two positive transcription factors. Intriguingly, the lack of direct binding of Smad3 to the *E2f8* promoter suggests that Smad3 could bind at other sites such as enhancers of the *E2f8* gene or could regulates of *E2f8* indirectly through other transcription factors, a fascinating question remaining to be elucidated in the future.

ChIP analysis also uncovered multiple binding domains for DBP and E2F8 upstream and downstream of the *Il9* promoter, to which these two transcription factors as well as Stat5a could directly bind. Importantly, TGF-β and IL-4 treatment enhanced the binding of DBP and Stat5a but removes the binding of E2F8 at the *Il9* promoter. Finally, both loss- and gain-of-function approaches have confirmed the activating and repressive functions of DBP and E2F8 respectively, for Th9 differentiation. Direct and acute knockdown of *Dbp* and *Stat5a* with their specific shRNAs significantly reduces IL-9 production, whereas knockdown of *E2f8* dramatically increases *Il9* gene expression. Conversely, overexpression of *Dbp* and *Stat5* in T cells significantly increases, but overexpression of *E2f8* suppresses, Th9 cell differentiation.

Recently, it was reported that IL-4 in combination with IL-1β also can activate Th9 differentiation in the absence of TGF-β signaling^54^. This study suggests that IL-1β/IL-4 induction IL-9 expression requires NF-κB but not TGF-β signaling. As IL-1β could induce TAK1 and NF-κB activation^55^, which is also critical for IL-9 expression^9^, it is likely that IL-lβ skips the TGF-β signaling to directly activate TAK1-NF-κB pathway and *Il9* gene expression. Although it remains to be elucidated, it is unlikely that IL-1β/IL-4-induced *Il9* gene expression is involved DBP and E2F8 pathways, because both transcription factors require TGF-β-pSmad3L-Ser^213^ signaling.

Finally and significantly, the opposing regulatory roles between DBP and E2F8 in Th9 cells contribute to Th9-mediated anti-tumor immunity in tumor in vivo and also play an important role in human TH9 differentiation. Supporting this conclusion are the findings obtained in two independent experimental models of melanoma and fibrosarcoma in mice. Reduction of *Dbp* and *E2f8* expression in CD4^+^ T cells respectively decreases and increases IL-9 production, and this decrease and increase respectively enhance and suppress tumor growth when the genetically engineered CD4^+^ T cells are adoptively transferred into the *Rag1*^*−/−*^ mice bearing the melanoma and fibrosarcoma. As expected, Stat5 in Th9 cells also has the anti-tumor effects in vivo. Intriguingly, inhibition of p38 with its inhibitor in Th9 cells did not show significant effects on the tumor growth compared to control Th9 cell treated tumor mice, despite the indisputable evidence that in vitro p38 inhibition significantly suppressed Th9 differentiation. The underlying mechanism remains unknown, but this might be because p38 has many other downstream targets and also affects other behavior of the T cells in vivo. The differential tumor immunotherapeutic effects are attributed to the changes in Th9 cells with *Dbp* and *E2f8* gene knockdown, as analysis of the tumor infiltrated lymphocytes (TIL) reveals no differences between the two groups with respect to other T cell subsets such as Th1, Th2, Th17 or Treg cells. As expected, and consistent with previous reports^8,9,43^, IL-9^+^ T cells are scarcely detectable in the TILs harvested from mice injected with Th9 cells, suggesting a dynamic and likely unstable feature of this subset^56^. However, the anti-tumor effect of transferred Th9 cells is indeed dependent on IL-9, as neutralization of IL-9 in the treated mice abolishes the antitumor function of the injected Th9 cells^7–9^. It has been suggested that Th9 cells initiate or incite a destructive program through secretion of granzyme B and other factors to cancer cells^11^. We revealed here that DBP and E2F8 in Th9 cells enhances and suppresses the expression of Granzyme B, respectively, in vitro and in vivo, but they have no significant effects on other anti-tumor cytokines, such as IL-3, IL-10, and IL-21. Moreover, it is also shown that Th9 may indirectly participate in anti-tumor immunity by activating other cells such as CD8^+^ T cells, mast cells, NK cells, ILC2s and by recruiting DCs^57^. We did not observe significant changes of mast cells and DCs in tumor tissues in *Dbp*- or *E2f8*-knockdowned Th9 cell treated tumor mice. This suggests that DBP and E2F8 in Th9 cells may function through regulation of Granzyme B for their anti-tumor function^43,58^. It remains a fascinating question whether Th9 cells can transform into other effector cells or educate and expand other immune effector T cells which in turn harbor tumor-suppressive functions^56^, as well as the exact executionary molecular mechanisms of Th9-cell mediated inhibition and/or killing of tumor cells. It should be pointed out that the roles of DBP and E2F8 in Th9 cells in the pathogenesis of other Th9-associated diseases such as allergy and asthma remain unknown and await further study. The findings that human DBP and E2F8 are also respectively upregulated and downregulated by TGF-β plus IL-4 stimulation and act as enhancer and suppressor to human TH9 differentiation reveals previously unrecognized targets for the investigation of TH9-mediated diseases and tumor immunotherapy in human patients.

In sum, we have discovered a previously unrecognized pivotal role of phosphorylation of pSmad3L-Ser^213^ in controlling the expression and function of two newly identified transcription factors, DBP and E2F8, in Th9 differentiation, in response specifically to TGF-β and IL-4 signaling. The opposing functions of DBP and E2F8 as enhancer and repressor respectively, in Th9 cells contribute to Th9-medaited anti-tumor effects in experimental models of melanoma and fibrosarcoma and suggest their biological significance in cancer immunotherapy. The opposing functions of DBP and E2F8, which have been reproduced and confirmed in human TH9 cells, provide potential clinical targets for the development of Th9 cell-mediated immunotherapy for human cancers.

## Methods

### Mice

Male and female C57BL/6 mice (#000664), *Rag1*^*−/−*^ mice (#034159), *Ert2-cre*^+^ mice (#008463), and *Cd4-cre*^*+*^ mice (#022071) were obtained from The Jackson Laboratory. The generation of *Tgfbr1*^f/f^ mice has been described^59^. *Smad3*^*−/−*^ mice^33^, *Tgfbr1*^f/f^ *Cd4*-*cre*^+^ mice^60^ and *Tgfbr1*^*f/f*^ *Ert2-cre*^+^ *mice*^61^ (on a C57BL/6 background) were bred under specific pathogen-free conditions in the animal facility of National Institute of Dental and Craniofacial Research. *Tgfbr1*^*f/f*^ *Ert2-cre*^+^ mice were generated in house by crossing *Ert2*-cre^+^ mice with *Tgfbr1*^f/f^ mice, and the mice were treated with tamoxifen (1 mg/mouse) per day for 5 days to delete TβRI. Both sexes in the age of 8 to 10 weeks were used for all experiments. All mice were co-bred under the same conditions in a 12 h light/dark cycle-, temperature (23  ±  3 °C)- and humidity (range 40-60)-controlled room. All animal studies were performed according to US National Institutes of Health guidelines for the use and care of live animals and approved by the Animal Care and Use Committees of National Institute of Dental and Craniofacial Research.

### Antibodies, reagents and plasmids

The following antibodies for flow cytometry (Supplementary Table 1**)**: Fluorochrome-conjugated anti-mouse CD3 (17A2) AF780 APC, anti-mouse CD3 (OKT3) APC-eFluor450, anti-mouse CD4 (RM4-5) PerCP, anti-human CD4 (RPA-T4) FITC, anti-mouse CD8a (53-6.7) FITC, anti-mouse IL-10 (JES5-16E3) FITC, anti-mouse IL-13 (eBio13A) PE, anti-mouse IL-17 (eBio17B7) PE/Cyanine7, anti-mouse IFN-γ (XMG1.2) eFluor450, anti-mouse IL-4 (11B11) PE, anti-mouse Foxp3 (FJK-16s) Pacific blue, anti-mouse Granzyme B (NGZB) APC and isotype-matched control antibodies (rat immunoglobulin G1 (IgG1) κ-chain (R3-34), rat IgG2a (R35-95) and rat IgG2b (A95-1)) were from eBioscience. Fluorochrome-conjugated anti-mouse IL-9 (RM9A4) APC, anti-human IL-9 (MH9A4) PE, anti-mouse TNF (MP6-XT22) FITC and anti-mouse IL-4 (11B11) PE/Cyanine7 were from BioLegend. The live/Dead fixable dead cell stain kit was purchased from Invitrogen. Antibodies for Western blotting were from the following sources (Supplementary Table 1**)**: Phosphor-Smad3L-Thr^179^ (ab74062), phosphor-Smad3L-Ser^213^ (ab63403), phosphor-Smad3 C-ter (ab52903), Smad3 (ab75512), Dbp (ab227591) and E2f8 (ab109596) were from Abcam. Phosphor-JNK (4668), total-JNK (9252), phosphor-ERK1/2 (9101), total-ERK (9102), phosphor-p38 (9211), total-p38 (9212), phosphor-Stat5 (9351), total-Stat5 (94205), total-Stat6 (9362), GAPDH (5174), and horseradish peroxidase-conjugated anti-rabbit IgG (7074) and anti-mouse IgG (7076) were from Cell Signaling Technology. phosphor-Stat6 was from Santa Cruz (sc-11762). Recombinant mouse IL-4 (404-ML), mouse IL-12 (419-ML), mouse IL-6 (406-ML), mouse IL-2 (402-ML), human IL-4 (204-IL) and human TGF-β1 (240-B) were from R&D Systems. In indicated experiments, MEK/ERK inhibitor U0126 (Promega), JNK inhibitor II SP600125 (Calbiochem), and p38 inhibitor SB203580 (Promega) were used at 10 μM.

### Murine and human primary T cell isolation and culture

Spleen and lymph nodes were isolated from mice, and red blood cells (RBC) were removed by ACK buffer. Naïve CD4^+^ CD62L^+^ cells or CD8^+^ cells were purified by MACS magnetic bead cell sorting (Miltenyi Biotec) and were cultured at 37 °C at a density of 1 × 10^6^ cells per well in 24-well plates with plate-bound anti-mouse CD3 (1 μg/ml) plus soluble anti-mouse CD28 (1 μg/ml) in the presence of TGF-β1 (2 ng/ml) plus IL-4 (10 ng/ml). Human peripheral blood mononuclear cells (PBMCs) provided by healthy volunteers were obtained from the Department of Transfusion Medicine (DTM) of the US National Institutes of Health through their approved protocol number NCT000001846. Naïve CD4^+^ T cells were purified with a Human Naïve T Cell Isolation kit (Miltenyi Biotec) as instructed in the manual. The isolated cells were cultured with plate-bound anti-human CD3 (1 μg/ml) and soluble anti-human CD28 (1 μg/ml). After 3 days, these cells were incubated with a combination of TGF-β1 (2 ng/ml) and IL-4 (10 ng/ml).

### Real-time RT-PCR

Total RNA was derived from cultured cells with an RNeasy Mini kit (Qiagen), and cDNA was synthesized with a High Capacity cDNA Reverse Transcription kit (Applied Biosystems). Quantitative real-time PCR was performed according to the protocol of TaqMan gene expression master mix (Applied Biosystems) using a QuantStudio 3 Real-Time PCR Systems (Applied Biosystems) with the following primers: mouse *Il9* (Mm00434305), mouse *Stat5a* (Mm00839861), mouse *Dbp* (Mm00497539), mouse *E2f8* (Mm01204160), mouse *Grzmb* (Mm00442837), mouse *Hprt*, (Mm00446968), human *IL9* (Hs00914237), human *STAT5A* (Hs00559643), human *DBP* (Hs00609747), human *E2F8* (Hs00226635) and human *ACTB* (Hs99999903). Results were normalized to those of *Hprt* or *ACTB* mRNA. All data were quantified by the 2^−ΔΔCT^ method.

### RNA-mediated interference and overexpression

For siRNA-mediated knockdown, the cells were transfected with human *STAT5A*-, *DBP*- and *E2F8*-specific siRNA (Sigma-Aldrich) using Lipofectamine RNAiMAX (Invitrogen) for 24 h. The constructs of Mouse *Stat5a*, *Dbp* and *E2f8* short hairpin RNA (shRNA) expression vectors in pGFP-V-RS plasmid were purchased from Origene. For overexpression, pCMV6-*Stat5a* (Origene), pPM-C-HA-*Dbp* (Abcam) and Myc-DDK-tagged *E2f8* (Origene) were used. All plasmid DNAs were isolated using the QIAGEN Plasmid kits (Qiagen) following the manufacturer’s instructions.

### Generation of SMAD3 constructs with linker region mutation

Plasmids for FLAG-tagged Smad3 wild-type, S213A, EPSM and ΔSSVS were kindly provided from Dr. Ying E. Zhang (NCI, NIH). These plasmids in retroviral pLPCX vector were made by subcloning these cDNA fragments from pCMV5B encoding FLAG-tagged Smad3^62^. The DNA fragments were amplified and subcloned into pMSCV-IRES-GFP retroviral vector between the *Xho*I and *EcoR*I restriction enzyme sites.

### Transfection

Human embryonic kidney (HEK) 293 T cells were purchased from the ATCC and were kept as a frozen stock. For transfection, HEK 293 T cells (2 × 10^6^) were cultured in a 10 cm^2^ diameter cell culture dish for overnight, and transiently transfected with specific target vector and pCL-ECO packaging vector (Addgene) using TurboFect transfection reagent (Thermo Scientific). After 48 h post-transfection, the culture supernatant containing viruses was collected. Using these viral supernatants, T cells were spinfected with polybrene (5 μg/ml). The transfection medium was then removed, and the T cells were cultured for an additional 48 h in complete cell culture medium.

### Flow cytometry

The cells were stained with antibodies specific to the various surface molecules, fixed and permeabilized with Fixation/Permeabilization buffer solution according to the manufacturer’s protocol (eBioscience). For intracellular cytokine staining, cells were stimulated for 4 h at 37 °C with PMA (phorbol 12-myristate 13-acetate; 10 ng/ml), ionomycin (1 μg/ml) and GolgiPlug (1:1,000 dilution; BD Pharmingen), followed by staining with Fixation/Permeabilization buffer solution according to the manufacturer’s protocol (BD Bioscience). The cells were incubated in FACS buffer (0.5% BSA and 0.1% NaN_3_ in PBS) with fluorochrome-conjugated antibodies (all diluted 1:100) for 30 min on ice. Stained cells were analyzed on a FACS Fortessa (BD Bioscience) with FlowJo software v 10.7.1.

### Western blot assay

The proteins samples were extracted with RIPA lysis buffer. They were separated by SDS-PAGE and transferred onto polyvinylidene difluoride (PVDF) membranes (Milipore). The membranes were blocked with 5% nonfat milk in TBST (0.5 M NaCl, Tris-HCl, pH 7.5 and 0.5% Tween-20) for 1 h at room temperature and separately incubated with the primary antibodies (all antibodies used at a dilution of 1:1000) in 5% BSA in TBST overnight at 4 °C. The membranes were washed with TBST buffer and then incubated with horseradish peroxidase-conjugated secondary antibodies (1:2000 dilution) for 1 h. The results were visualized by enhanced chemiluminescence using an Amersham Imager 600 (Cytiva) according to the manufacturer’s protocol.

### ELISA

For IL-9 cytokine assays, the cell culture supernatants were collected after 72 h. Cytokine was quantified using IL-9 ELISA kit (BioLegend) according to the manufacturer’s protocol.

### DNA-Seq assay

Cells were crosslinked with 1% formaldehyde at room temperature for 10 min. 0.3 unit of DNase I (Roche) was added to the cells and incubated at 37 °C for 5 min. The reaction was stopped by adding 10 mM EDTA. The DNase-seq libraries were prepared using Illumina kits, and were amplified preferentially using a two-step method to amplify the short DNA fragments released from the DNA I hypersensitive sites. The fragments were isolated on E-gel SizeSelect agarose gels (Invitrogen), and the libraries were sequenced on the Illumina HiSeq 2500.

### RNA-seq assay

Cells were sorted directly into 700 μl of QIAzol Lysis Reagent in the miRNAeasy Micro Kit (QIAGEN). Total RNA was extracted and on-column digestion with DNase (QIAGEN) was performed, followed by elution with RNase-free water, then were reverse transcribed by SuperScript II (Invitrogen). cDNA was pre-amplified by PCR using KAPA HiFi HotStart ReadyMix (Kapa Biosystems, 2602), and purified using Ampure XP beads (Beckman Coulter). RNA-seq libraries were prepared through the Smart-seq2 method^63^. We performed GO analysis for 12 differential clusters (Supplementary Data 2) using the category of biological process in Gene Ontology Consortium. The color level represents the negative logarithm of the adjusted *p*-value (*p*-value of 0.05) for the significance of the GO terms. The intensity of red represents greater significance of the GO terms whereas blue represents less significant GO terms. Benjamini and Hochberg correction for multiple tests.

### Processing of the sequencing data

Reads from DNase-seq and RNA-seq data were mapped to mouse genome (mm9) using Bowtie2. Reads with MAPQ ≤ 10 or redundant reads that mapped to the same location and orientation were removed. DNase-seq data was normalized by the total library size. DHS from DNase-seq data were called by MACS as ‘narrow peaks’. Gene expression level was then measured by calculating the normalized reads per kilobase per million mapped reads (rpkm) for each gene using EdgeR (FDR < 0.01 and fold-change > 1.5).

### Motif finding

FIMO was applied to the differential DNase hypersensitive sites in order to identify the enriched motifs (*p*-value < 0.001).

### Chromatin immunoprecipitation (ChIP) assay

ChIP assay was carried out by using a ChIP assay kit (Diagenode). Briefly, CD4^+^ T cells were cultured at a density of 5 × 10^6^ cells per well in 6 well plates with anti-CD3 and anti-CD28 in the presence or absence of TGF-β1 (2 ng/ml) and IL-4 (10 ng/ml) for 1 h. The cells were collected and then were fixed with 1% formaldehyde and lysed in lysis buffer for 10 min at room temperature. Lysates were sonicated using the Bioruptor Standard (Diagenode), followed by precipitation with anti-Smad3 and control rabbit IgG. One-tenth of each chromatin sample was kept as the input DNA control. ChIP and total input DNAs were analyzed by real-time RT-PCR with SYBR green PCR reagent (Qiagen) using the following primers for *Stat5a*, *Dbp*, *E2f8* and *Il9* promoter (Supplementary Table 2).

### Tumor-bearing model

CD4^+^ naïve T cells were isolated from C57BL/6 mice and were cultured with anti-CD3 and anti-CD28 overnight, then control shRNA, *Stat5* shRNA, *Dbp* shRNA or *E2f8* shRNA was added. After 48 h, the cells were continued being cultured with TGF-β1 and IL-4 for another 3 days for cell transfer. B16F10 melanoma cells or MCA205 fibrosarcoma cells (5 × 10^5^ cells per mouse) were injected subcutaneously at the side of the abdomen of male and female *Rag1*^*−/−*^ mice to set up tumor-bearing model. On the same day, the cultured T cells were transferred into *Rag1*^*−/−*^ mice intravenously (1 × 10^7^ cells per mouse). To check the anti-tumor therapeutic effects of *E2f8*-knockdown Th9 cells in vivo, the Th9 cells were injected intraperitoneally with IgG2a isotype control or anti-IL-9 antibody in tumor-bearing mice injected with *E2f8*-knockdowned T cells (100 µg per mice, every 3 days). Tumor size was measured in two perpendicular dimensions with calipers every 2 or 3 days, then calculated tumor volume using formulations Volume = (tumor length x tumor width^2^)/2. Two weeks after we implant tumor cells, the mice were euthanized when tumor size reaches 20 mm in any mice in any group, and we collected tissues to analyze the change in tumor tissue milieu.

### Statistical analysis

Data were analyzed and represented graphically using GraphPad software Prism 8. Mean ± standard error of the mean (SEM) of at least three independent experiments were calculated for all experiments. The unpaired two-tailed Student’s *t*-test was used for the comparison of means between two independent groups; and the one-way ANOVA was used for the comparison of means between two or more groups. If the *p* value was less than 0.05, the differences were considered to be statistically significant.

### Reporting summary

Further information on research design is available in the Nature Research Reporting Summary linked to this article.

## Supplementary information

Supplementary Information

Description of Additional Supplementary Files

Supplementary Data 1

Supplementary Data 2

Supplementary Data 3

Reporting Summary

## Data Availability

All array data support the findings of this study have been deposited in the NCBI Gene Expression Omnibus database under accession code GSE182472. The authors declare that all other data supporting the findings of this study are available within the article and its supplementary information files. Source data are provided with this paper.

## Source data

Source Data
