# Supplementary material for: Opposing functions of circadian protein DBP and atypical E2F family E2F8 in anti-tumor Th9 cell differentiation
Source: Nat Commun. 2022 Oct 14;13:6069. doi: 10.1038/s41467-022-33733-8 (PMC9568563; doi:10.1038/s41467-022-33733-8)
Supplement: Supplementary file 1 — Supplementary Information [file 41467_2022_33733_MOESM1_ESM.pdf]

Supplementary Information

Opposing functions of two transcription factors, DBP and E2F8, in anti-tumor Th9 cell differentiation

Sang-A Park[1#+], Yun-Ji Lim[1#], Wai Lim Ku[2], Dunfang Zhang[1], Kairong Cui[2], Liu-Ya Tang[3], Cheryl Chia[1], Peter Zanvit[1], Zuojia Chen[4], Wenwen Jin[1], Dandan Wang[1], Junji Xu[1], Ousheng Liu[1], Fu Wang[1], Alexander Cain[1], Nancy Guo[1], Hiroko Nakatsukasa[1], Chuan Wu[4], Ying E. Zhang[3], Keji Zhao[2*] and WanJun Chen[1*]

[1]Mucosal Immunology Section, National Institute of Dental and Craniofacial Research, National Institutes of Health, 30 Convent Drive, Bethesda, MD 20892, USA.

[2]Systemic Biology Center, National Heart, Lung, and Blood Institute, National Institutes of Health, 31 Center Drive, Bethesda, MD 20892, USA.

[3]Laboratory of Cellular and Molecular Biology, Center for Cancer Research, National Cancer Institute, National Institutes of Health, 37 Convent Drive, Bethesda, MD 20892, USA.

[4]Experimental Immunology Branch, National Cancer Institute, National Institutes of Health, 37 Convent Drive, Bethesda, MD 20892, USA.

[#]These authors contributed equally: Sang-A Park, Yun-Ji Lim

[+]Deceased

[*]Corresponding to K.Z. zhaok@nhlbi.nih.gov; W.J.C. wchen@mail.nih.gov

Supplementary Figure 1-10 and legends

Supplementary Table 1-2

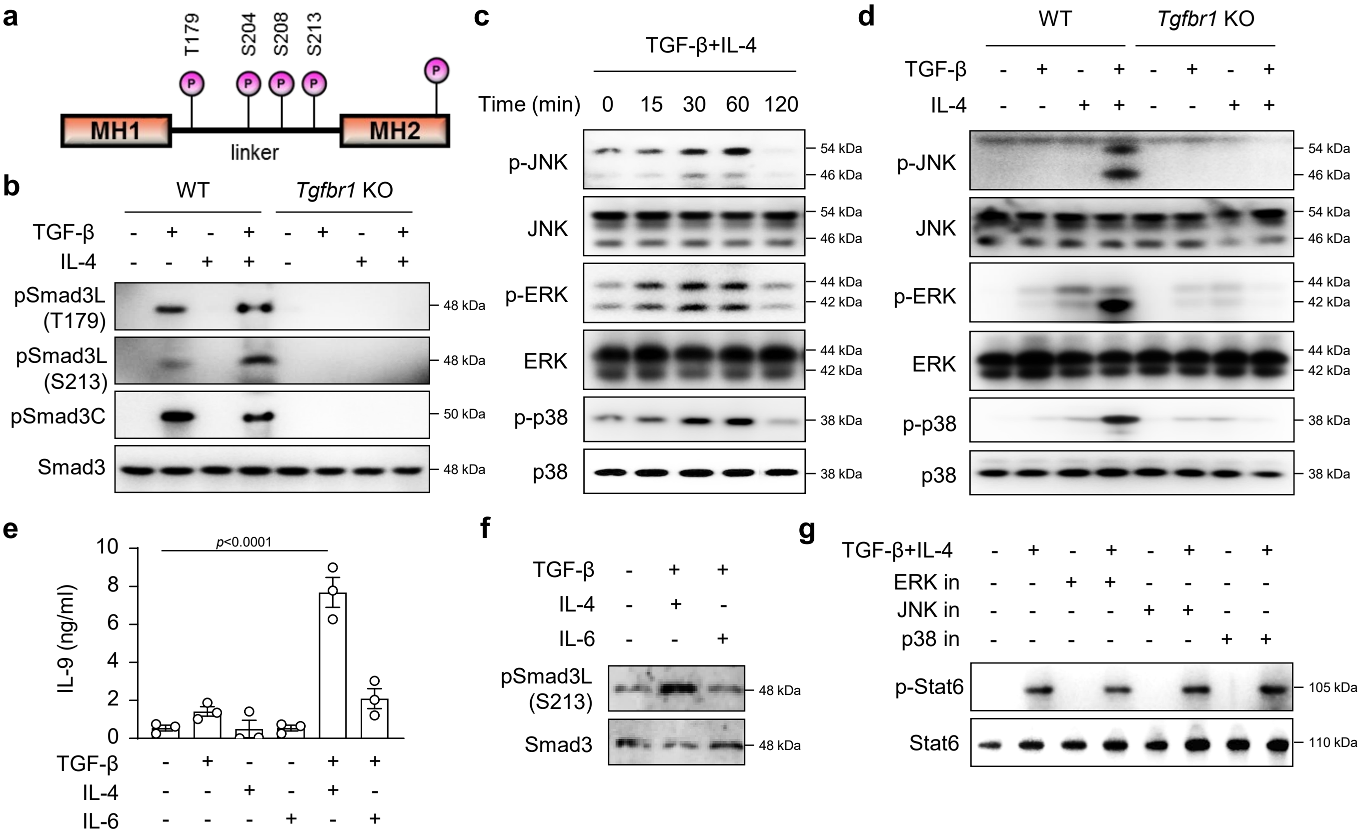

Supplementary Figure 1. TGF-β plus IL-4 induce activation of MAPK

a Schematic depiction of Smad3 linker region. **b** Western blot analysis of phosphorylated linker region Thr^{179}, Ser^{213} sites and C-terminal of Smad3 and total Smad3 in WT and *Tgfbr1* KO CD4^{+} naïve T cells cultured with medium, TGF-β, IL-4 or TGF-β plus IL-4 for 1 h. **c** Time course of MAPK (ERK, JNK and p38) activation of CD4^{+} T cells in response to TGF-β and IL-4. **d** MAPK phosphorylation in WT and *Tgfbr1* KO CD4^{+} T cells cultured with TGF-β, IL-4 or TGF-β plus IL-4 for 1 h. **e** Measurement of IL-9 in culture supernatants of CD4^{+} T cells in the presence of TGF-β, IL-4, IL-6, TGF-β plus IL-4 or TGF-β plus IL-6 for 72 h. **f** Western blotting of phosphorylated Smad3L-Ser^{213} in response to TGF-β plus IL-4 or TGF-β plus IL-6 in CD4^{+} T cells cultured for 2 h. **g** Western blotting of Stat6 phosphorylation in T cells pre-treated with MAPK inhibitors and then cultured with TGF-β plus IL-4 for 2 h. **e** The data were by one-way ANOVA with Tukey's test. **b-e** The data were representative of three independent experiments or **f,g** two experiments. Graphs show the mean ± SEM. Source data are provided as a Source Data file.

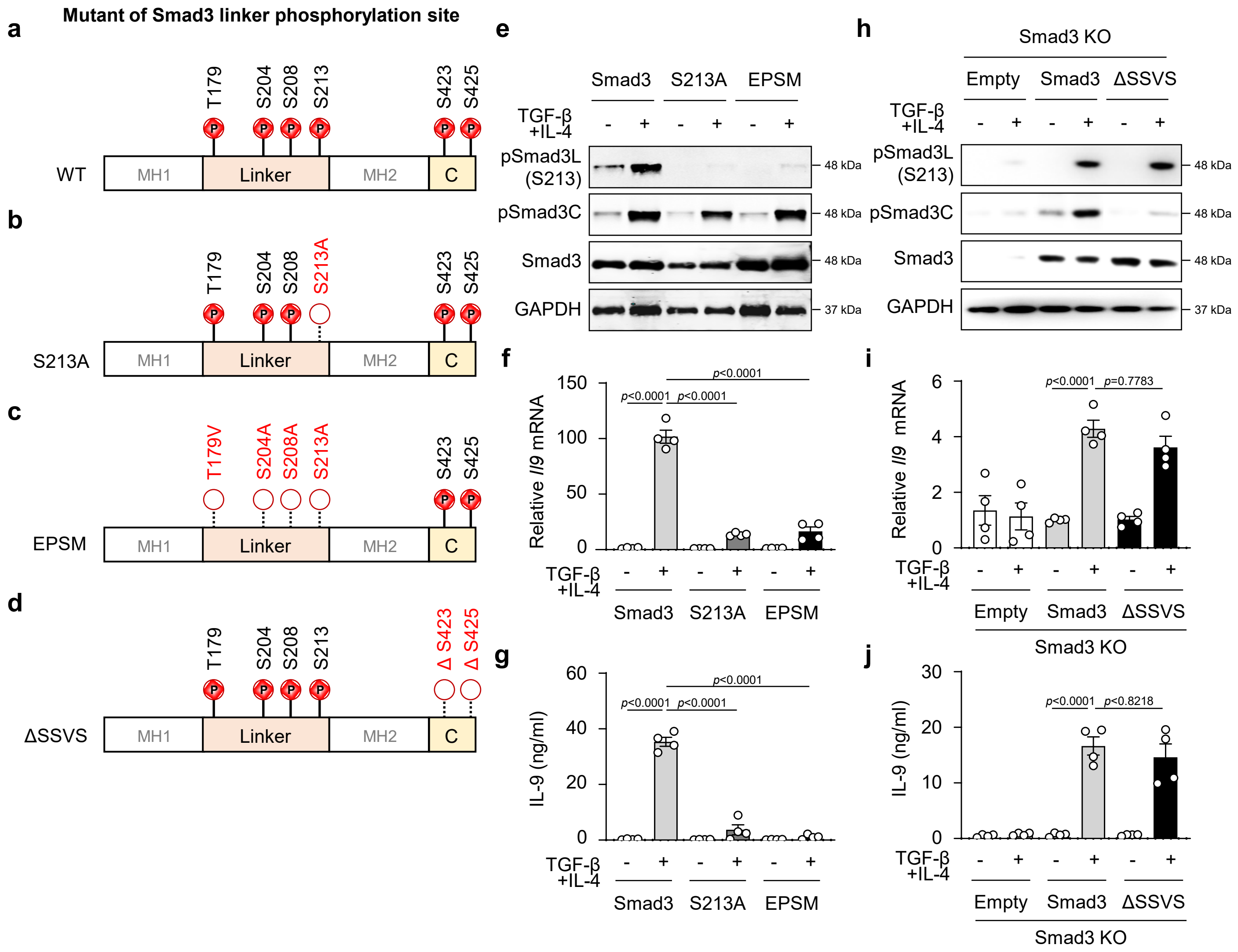

Supplementary Figure 2. pSmad3 is dispensable for IL-9 production induced by TGF-β and IL-4

a-d Schematic illustration of WT Smad3, S213A, EPSM mutants at the linker region and ΔSSVS mutants of Smad3 at the C-terminal phosphorylation sites. **e,h** Western blotting of phosphorylated Smad3 by TGF-β plus IL-4 for 2 h in WT Smad3-, S213A- or EPSM-transfected WT $CD4^+$ T cells **e** and WT Smad3- or ΔSSVS-transfected $Smad3^{-/-}$ T cells **h**. **f,i** *Il9* mRNA after 24 h by real-time RT-PCR and **g,j** secreted IL-9 in the culture supernatants by ELISA in T cells transfected with indicated Smad3 mutants as in **e** and **h** after 72 h. Alanine (A), Aspartic acid (D), Serine (S), Threonine (T) and Valine (V), respectively. These data were analyzed by one-way ANOVA with Tukey's test. **e,h** These data were representative of three independent experiments, or **f,g,l,j** four experiments. Graphs show the mean ± SEM. Source data are provided as a Source Data file.

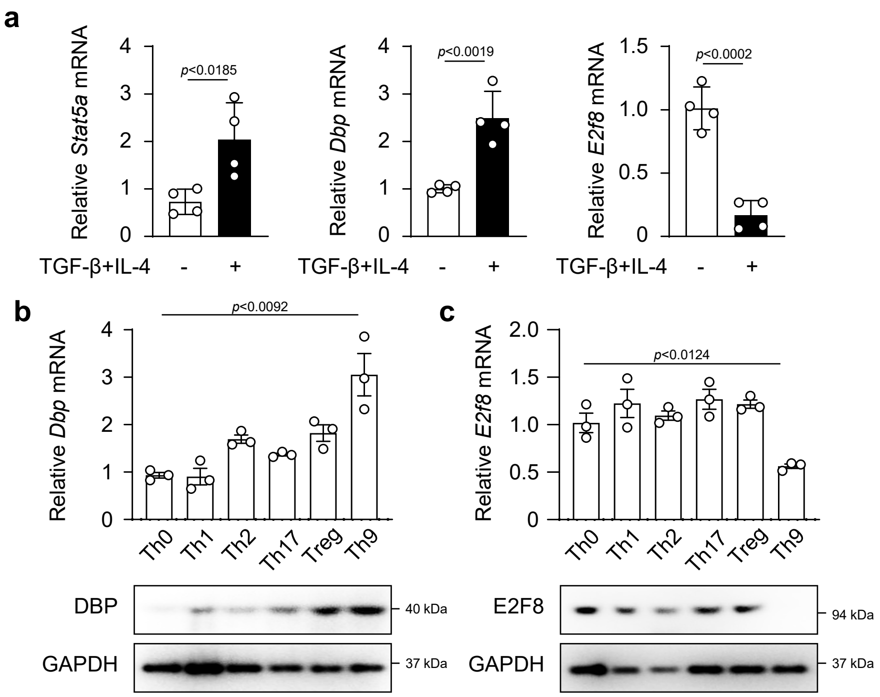

Supplementary Figure 3. Expression of DBP and E2F8 in other T cell subsets

a Gene expression of *Stat5a*, *Dbp* and *E2f8* in naïve CD4+ T cells followed by stimulation with TGF-β plus IL-4 for 3 days. **b** DBP and **c** E2F8 expression in CD4+ T cells in the presence of IL-12 (10 ng/ml); Th1, IL-4 (10 ng/ml); Th2, TGF-β (2 ng/ml) plus IL-6 (25 ng/ml); Th17, TGF-β (2 ng/ml) plus IL-2 (10 ng/ml); Treg or TGF-β (2 ng/ml) plus IL-4 (10 ng/ml); Th9. These data were analyzed by two-tailed unpaired Student's *t*-test. **a** The data were representative of four independent experiments or **b,c** three experiments. Graphs show the mean ± SEM. Source data are provided as a Source Data file.

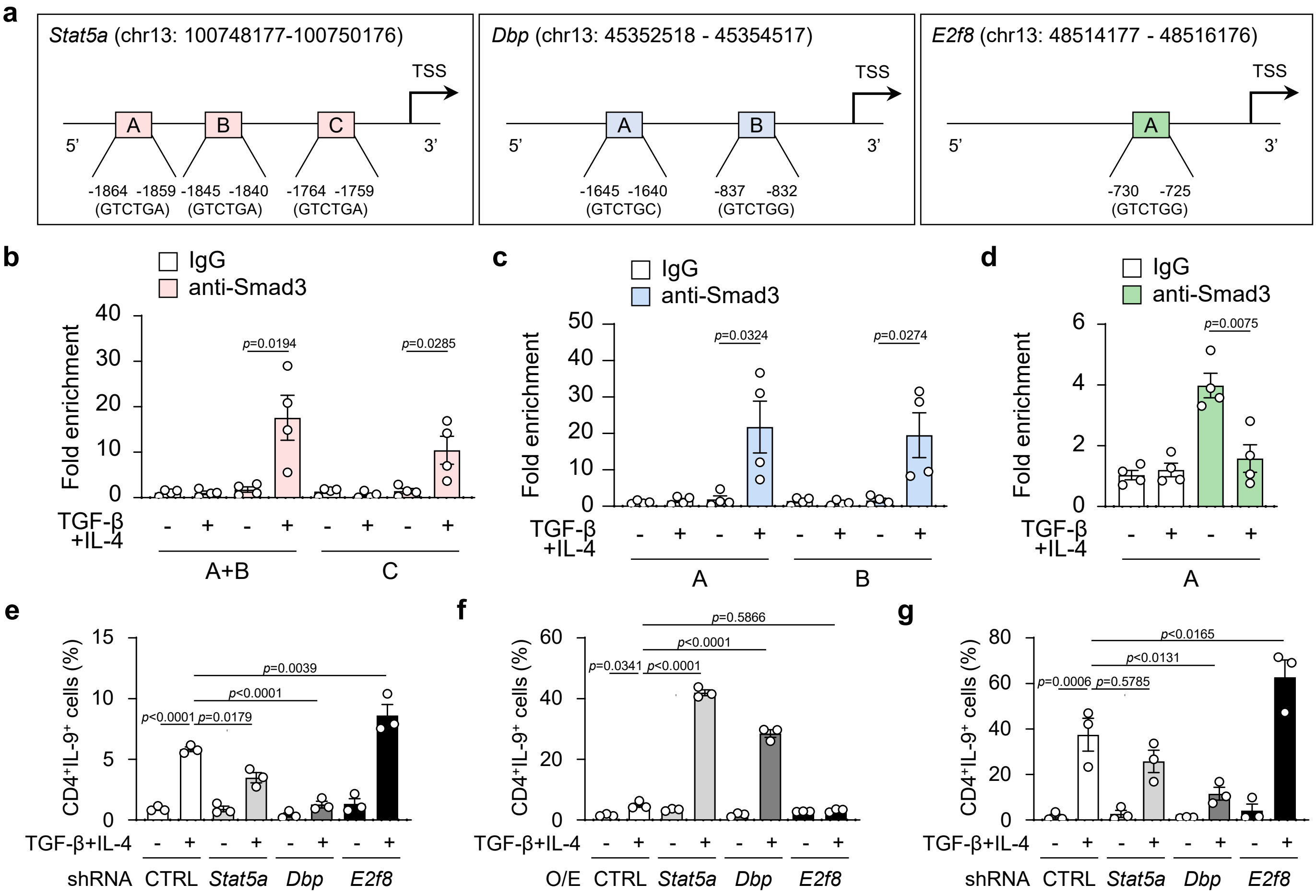

Supplementary Figure 4. Smad3 binding at *Stat5a*, *Dbp* and *E2f8* during Th9 differentiation

a Schematic representation of Smad3 binding sites on *Stat5a*, *Dbp* and *E2f8* promoter region. **b-d** $CD4^+$ T cells incubated with or without TGF-β plus IL-4 for 1 h and subjected to ChIP assays with Smad3 antibody and PCR primers. **e,f** Intracellular staining of IL-9 and in $CD4^+$ T cells transfected with *Stat5a*, *Dbp* or *E2f8* specific shRNA **e** or overexpressing virus **f**, followed by stimulation with TGF-β plus IL-4 for 3 days. **g** Intracellular staining of IL-9 in **e** for 5 days. O/E, Overexpressed. **b-d** The data were analyzed by two-tailed unpaired Student's *t*-test or **e-g** one-way ANOVA with Tukey's test. **b-d** The data were representative of four independent experiments or **e-g** three experiments. Graphs show the mean ± SEM. Source data are provided as a Source Data file.

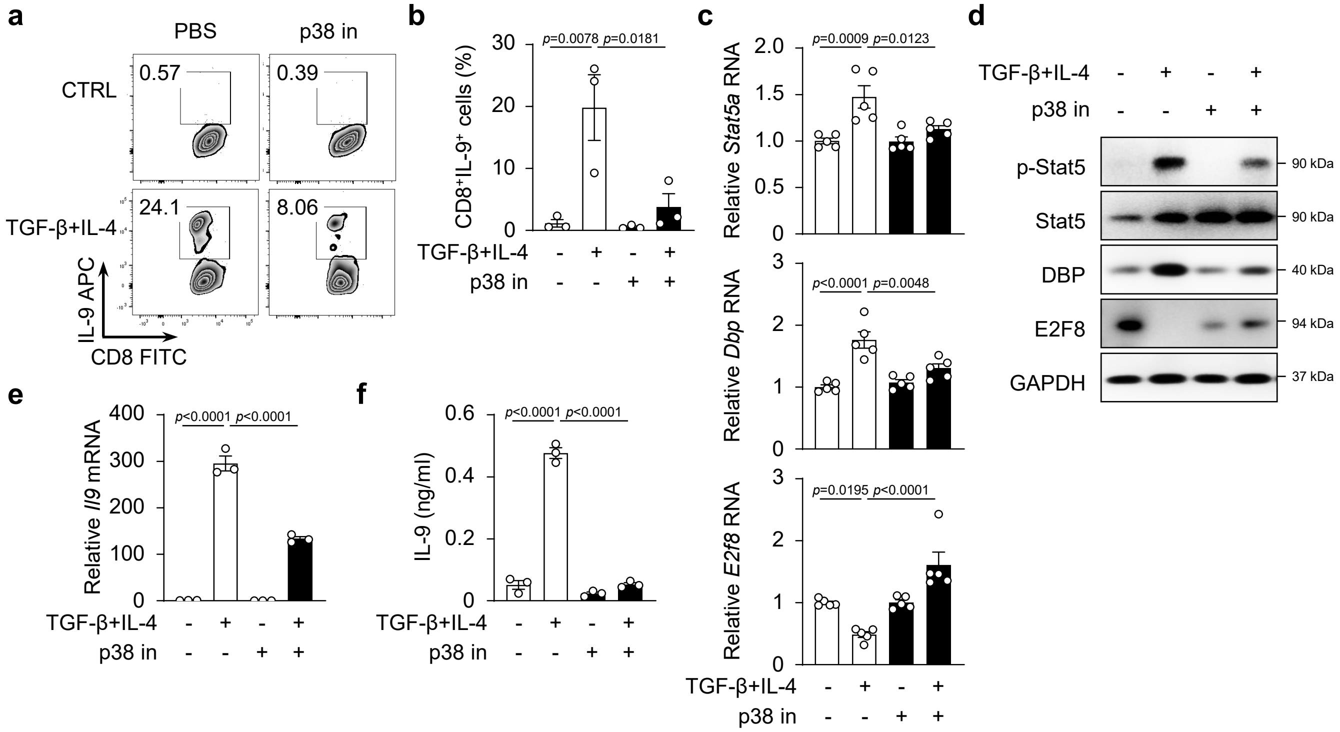

Supplementary Figure 5. IL-9 production of Tc9 via TGF-β plus IL-4 requires DBP and E2F8

a Intracellular staining of IL-9 by flow cytometry in CD8+ T cells stimulated with TGF-β plus IL-4 in the presence or absence of the p38 inhibitor for 72 h. **b** Summary of results in **a**. **c** RT-PCR analysis and **d** Western blotting of Stat5a, Dbp and E2f8 in CD8+ T cells cultured with TGF-β plus IL-4 in the presence or absence of the p38 inhibitor for 24 h **c** and 72 h **d** as in **a**. **e** Expression of IL-9 mRNA and **f** secreted protein of cells after 72 h as in **a**. These data were analyzed by one-way ANOVA with Tukey's test. **a,b,d-f** The data were representative of three independent experiments or **c** five experiments. Graphs show the mean ± SEM. Source data are provided as a Source Data file.

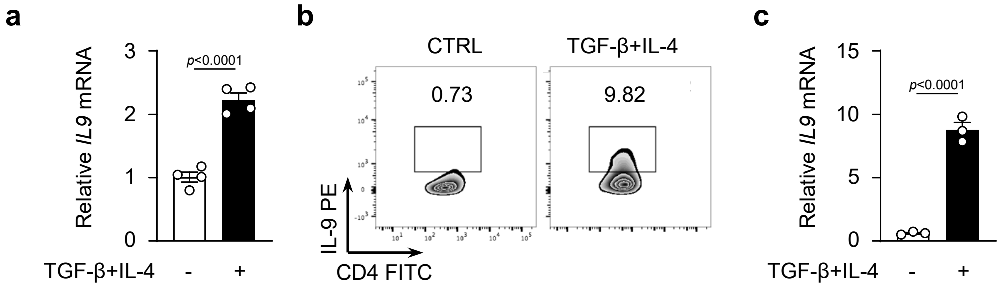

Supplementary Figure 6. IL-9 production of human TH9 in response of TGF-β plus IL-4

a Expression of *Il9* mRNA in human CD4^{+} T cells from peripheral blood mononuclear cells in the absence and presence of TGF-β and IL-4 for 3 days. **b** Intracellular staining of IL-9 in human CD4^{+} T cells stimulated in a for 5 days. **c** Summary of results in **b**. The data were analyzed by two-tailed unpaired Student's *t*-test. **a** These data were representative four independent experiments or **b,c** three experiments. Graphs show the mean ± SEM. Source data are provided as a Source Data file.

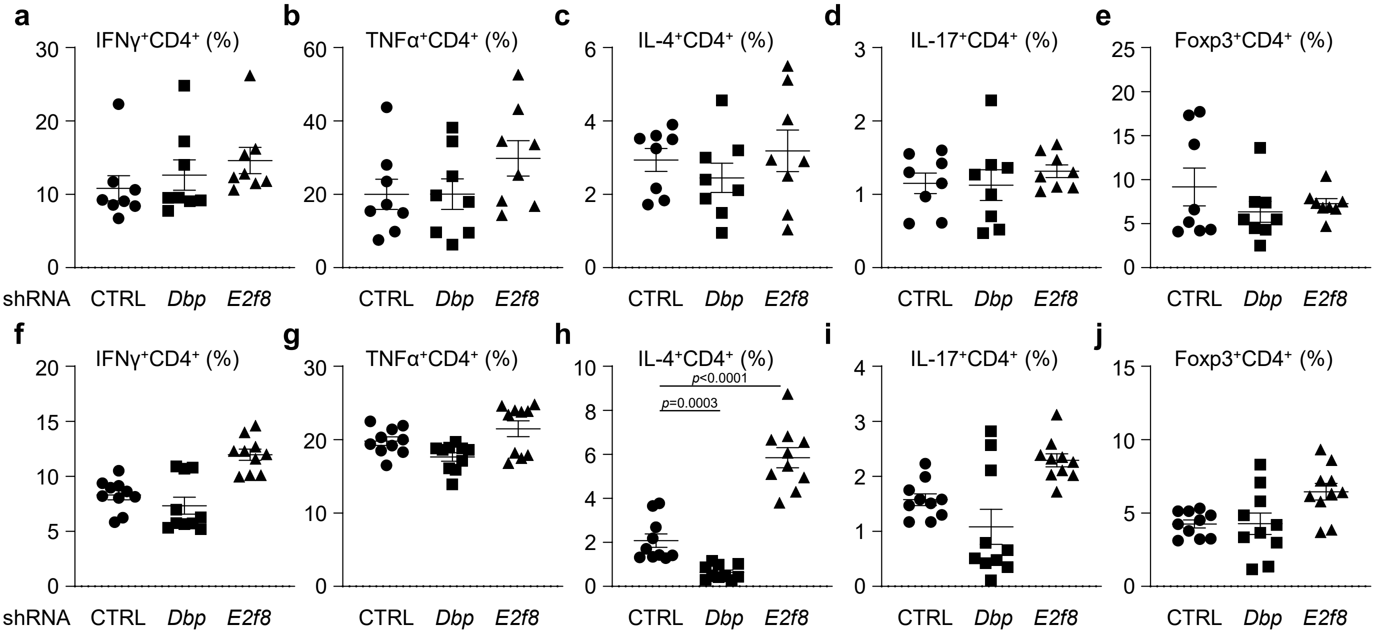

Supplementary Figure 7. Analyses of Th1, Th2, Th17 and Treg cells in the tumors of mice treated by indicated Th9 subsets

Intracellular staining of IFN-γ, TNF-α, IL-4, IL-17 and Foxp3 in intratumoral $CD4^+$ T cells. **a-e** A group of 8-week-old male (n=5) and female (n=3) *Rag1*$^{-/-}$ mice was injected with B16 melanoma (n=8 per group). **f-j** A group of 8-week-old male (n=5) and female (n=5) *Rag1*$^{-/-}$ mice was injected with MCA205 fibrosarcoma (n=10 per group). Data were analyzed by two-tailed unpaired Student's *t*-test. These data were presented by two independent experiments. Graphs show the mean ± SEM. Source data are provided as a Source Data file.

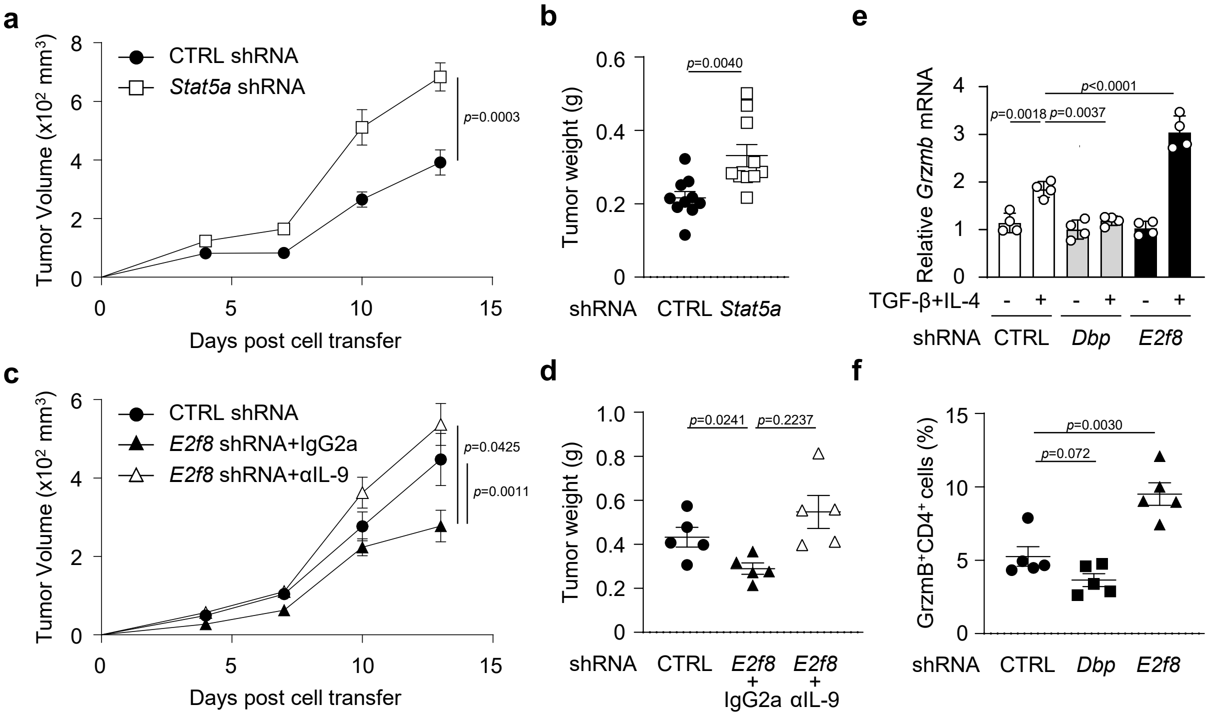

Supplementary Figure 8. Anti-tumor effect of Th9 cells through the granzyme B.

a,b A group of 8-week-old male (n=5) and female (n=5) *Rag1*$^{-/-}$ mice were injected with MCA205 fibrosarcoma, and *CTRL* or *Stat5* shRNA-treated Th9 cells (n=10 per group). **c,d** *Rag1*$^{-/-}$ mice were injected with MCA205 fibrosarcoma,and *E2f8* shRNA-treated Th9 cells (male: n=5 per group), then they were injected intraperitoneally with IgG2a isotype or anti-IL-9 antibody (100 µg per mice, every 3 days). **a,c** Tumor growth of these mice was measured every 3 days, and **b,d** tumor weight at the end of the experiments. **e** *Granzyme b* mRNA expression in CD4^{+} T cells transfected with *Dbp* or *E2f8* shRNA, followed by stimulation with TGF-β plus IL-4 for 3 days *in vitro.* **f** Granzyme B production. A group of 8-week-old male mice were injected with MCA205 fibrosarcoma (n=5). **a-d,f** These data were analyzed by two-tailed unpaired Student's *t*-test or **e** one-way ANOVA with Tukey's test. **a,b** These data were representative of two independent experiments, **c,d,f** a single experiment or **e** four experiments. Graphs show the mean ± SEM. Source data are provided as a Source Data file.

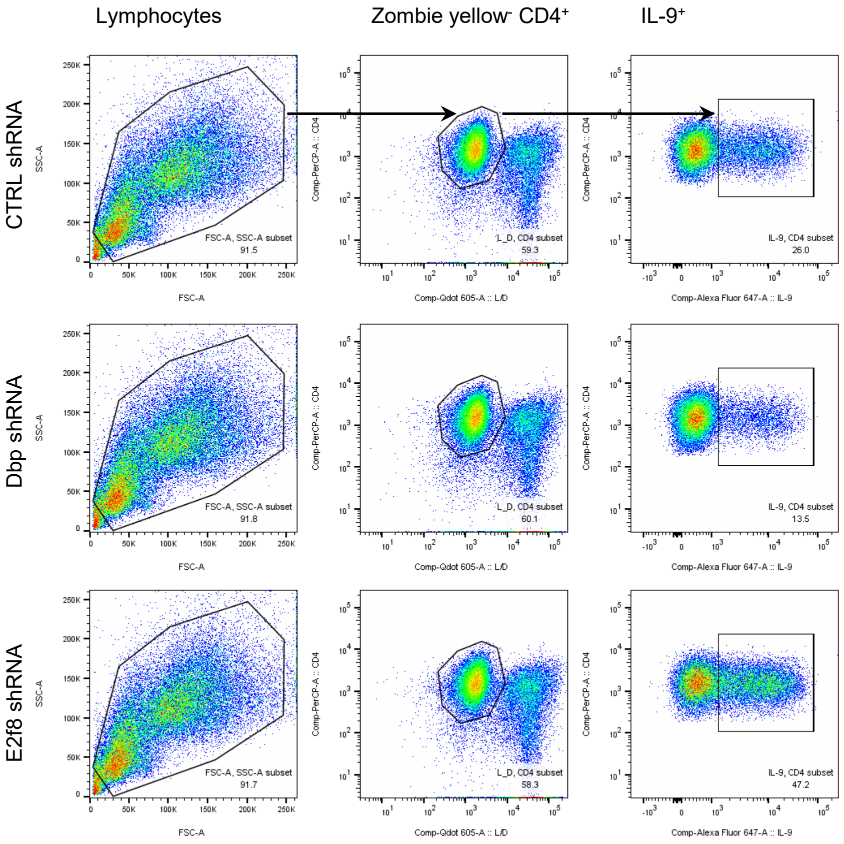

Supplementary Figure 9. Gating strategy to determine IL-9⁺ CD4⁺ T cells.

Gating strategy to determine IL-9-producing Th9 (Zombie yellow⁻CD4⁺IL-9⁺) cells transfected with *Stat5a*, *Dbp* or *E2f8* specific shRNA as in **Fig. 6a**.

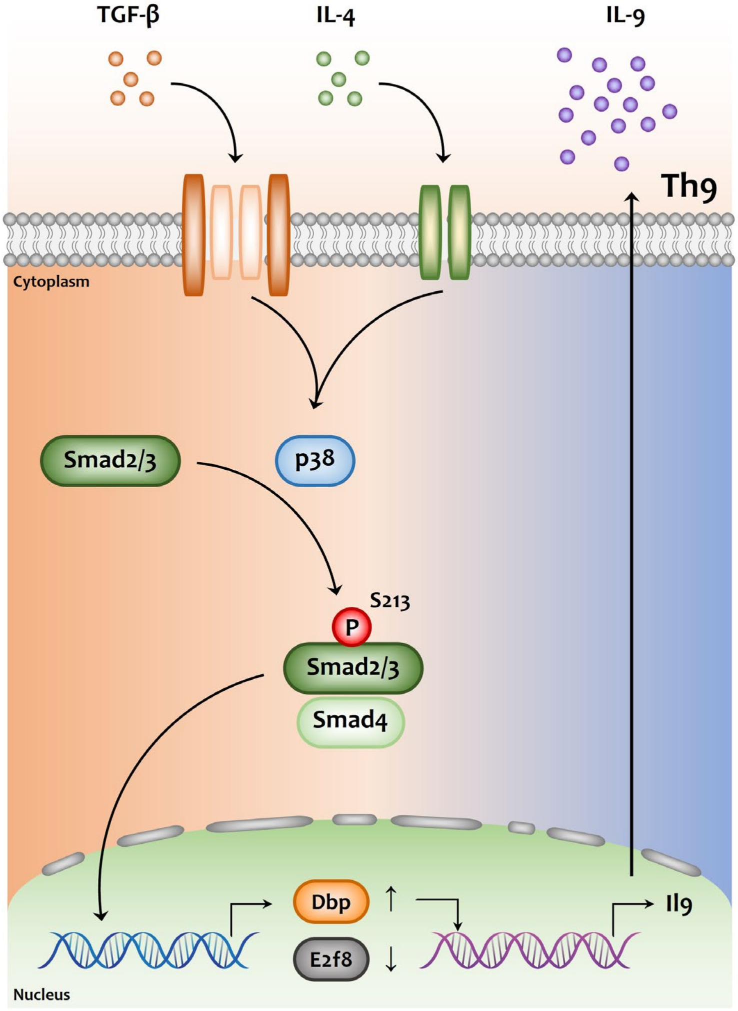

Supplementary Figure 10. TGF-β and IL-4 signaling induce phosphorylation of the Serine 213 site in the linker region of Smad3 protein (pSmad3L-Ser^{213}) via the activation of p38 in naïve CD4^{+} T cells. pSmad3L-Ser^{213} is essential and sufficient for Th9 differentiation. The phosphorylation of other sites such as Thr^{179} in the linker region or C-terminal region of Smad3 in however dispensable for Th9 differentiation induced by TGF-β and IL-4. Using DNA-seq and RNA-seq analyses in CD4^{+} T cells stimulated with TGF-β and IL-4 in comparison to T cells treated with TCR stimulation alone and to TGF-β receptor I deficient T cells treated with TGF-β and IL-4, we identify that Dbp is upregulated and E2f8 downregulated in TGF-β and IL-4 treated T cells. The changes of Dbp and E2f8 are dependent on pSMAD3L-Ser^{213} during TGF-β and IL-4 signaling. We reveal that transcription factors Dbp and E2f8 as key activator and repressor for *Il9* gene expression during Th9 cell differentiation.

Supplementary Table 1. Antibodies list for flow cytometry and western blot

Antibodies for FACS

Antibody	Clone	Reactivity	Supplier	Fluorophore	Catalog No.	Dilution
CD4	RM4-5	Mouse	eBioscience	PerCP	45-0042-82	1:100
CD3	OKT3	Mouse	eBioscience	APC-eFluor450	48-0037-42	1:100
CD3	17A2	Mouse	eBioscience	AF780 APC	47-0032-82	1:100
CD8a	53-6.7	Mouse	eBioscience	FITC	11-0081-82	1:100
IL-9	RM9A4	Mouse	BioLegend	APC	514106	1:100
IL-4	11B11	Mouse	eBioscience	PE	12-7041-82	1:100
IL-10	JES5-16E3	Mouse	eBioscience	FITC	17-7101-82	1:100
IL-13	eBio13A	Mouse	eBioscience	PE	12-7133-82	1:100
IL-17	eBio17B7	Mouse	eBioscience	PE/Cyanine7	25-7177-82	1:100
IFNr	XMG1.2	Mouse	eBioscience	eFluor450	48-7311-82	1:100
TNF	MP6-XT22	Mouse	BioLegend	FITC	506304	1:100
Foxp3	FJK-16s	Mouse	eBioscience	Pacific Blue	48-5773-82	1:100
Granzyme B	NGZB	Mouse	eBioscience	APC	17-8898-82	1:200
CD4	RPA-T4	Human	eBioscience	FITC	11-0049-42	1:100
IL-9	MH9A4	Human	BioLegend	PE	507605	1:100

Antibodies for western blot

Antibody	Size (kDa)	Supplier	Catalog No.	Dilution	Host
p-Smad3L T179	48	Abcam	ab74062	1:1000	Rabbit
p-Smad3L S213	48	Abcam	ab63403	1:1000	Rabbit
p-Smad3C	50	Abcam	ab52903	1:1000	Rabbit
Smad3	48	Abcam	ab75512	1:1000	Mouse
p-SAPK/JNK	54, 46	Cell signaling	4668	1:1000	Rabbit
JNK	54, 46	Cell signaling	9252	1:1000	Rabbit
p-ERK1/2	44, 42	Cell signaling	9101	1:1000	Rabbit
ERK1/2	44, 42	Cell signaling	9102	1:1000	Rabbit
p-p38	38	Cell signaling	9211	1:1000	Rabbit
p38	38	Cell signaling	9212	1:1000	Rabbit
p-STAT5	90	Cell signaling	9351	1:1000	Rabbit
STAT5	90	Cell signaling	94205	1:1000	Rabbit
DBP	40	Abcam	ab227591	1:1000	Rabbit
E2F8	94	Abcam	ab109596	1:1000	Rabbit
p-STAT6	105	Santa Cruz	sc-11762	1:1000	Rabbit
STAT6	110	Cell signaling	9362	1:1000	Rabbit
GAPDH	37	Cell signaling	5174	1:2000	Rabbit
Rabbit IgG		Cell signaling	7074	1:2000	
Mouse IgG		Cell signaling	7076	1:2000	

Supplementary Table 2. Sequences of primers used for ChIP-qPCR analysis

Primer name	Sequence
Stat5a-sm3 1f	CCAGGGCCAAGATTACAAAGTA
Stat5a-sm3 1r	CGGACAGTGCTTCTTCTCTTC
Stat5a-sm3 2f	GGGAAGAGAAGAAGCACTGTC
Stat5a-sm3 2r	AACTGAATTCGGAGGGAAAGG
Dbp-sm3 1f	CAGTCTCCATACGACAACAGAC
Dbp-sm3 1r	GTCACCTGGAGGGAGGTAATA
Dbp-sm3 2f	GGTCACCTGTTTACAAATGGTATG
Dbp-sm3 2r	TGCATAAAGGTACATGGAGAGG
E2f8-sm3 1f	TAGGAGGTGGAGCCTGTTTA
E2f8-sm3 1r	AACAACTTTGGCAGTCCCT
Il9-stat5a ds f	GGTCTGGGGTGTGATTTTGC
Il9-stat5a ds r	AAACGGAAAAGGGAGCCATCT
Il9-stat5a 1f	TCCCACTCATTCGTCTGTCTG
Il9-stat5a 1r	CCTTTCTGACCTCTGGAGCA
Il9-stat5a 2f	GGTGGCTCTTACCAGATGTCA
Il9-stat5a 2r	TGTGTAGACGCAGACAAAGACA
Il9-stat5a 3f	ACACCGCCATAGACCTGTCA
Il9-stat5a 3r	TCAGACCACAGGGGCTTGAA
Il9-stat5a 4f	AAAAACAGCTGCTTGGCAGA
Il9-stat5a 4r	ATCCAGCAAGTTTCCAGTAAACAG
Il9-dbp ds f	GTGTGAACGTTCTTGGGCTTT
Il9-dbp ds r	ATCCAGCAAGCAAAGGTCAG
Il9-dbp 1f	TTTCTAGGCAGTGCTGCGAT
Il9-dbp 1r	GGGACTCGAATGCAAGGCTA
Il9-dbp 2f	CTCCTAAGCCTTACAAGAAAGT
Il9-dbp 2r	CAATCAACAAGCCTCAGTAAAC
Il9-dbp 3f	CAGACAAGCCTCATGACATCC
Il9-dbp 3r	ATCCAGCAAGTTTCCAGTAAACAGT
Il9-e2f8 ds f	GGGGTGTGATTTTGCCACTTG
Il9-e2f8 ds r	GTCACCTCCAAACGGAAAAGG
Il9-e2f8 1f	CCTAGTGACACTTATCGGCCT
Il9-e2f8 1r	CTCCTTTCTGACCTCTGGAGC
Il9-e2f8 2f	GTCTACACCGCCATAGACCTG
Il9-e2f8 2r	ACCACAGGGGCTTGAAGATTA
