# Supplementary material for: Opposing functions of circadian protein DBP and atypical E2F family E2F8 in anti-tumor Th9 cell differentiation
Source: Nat Commun. 2022 Oct 14;13:6069. doi: 10.1038/s41467-022-33733-8 (PMC9568563; doi:10.1038/s41467-022-33733-8)
Supplement: Supplementary file 2 — Description of Additional Supplementary Files [file 41467_2022_33733_MOESM2_ESM.pdf]

Description of Additional Supplementary Files

Supplementary Data 1. The gene expression levels in CD4+ T cells as determined by RNA-seq. Description in main text and in the RNA-seq data processing.

Supplementary Data 2. Gene list in the 12 clusters.

Supplementary Data 3. The comparison between the gene expression between WT TGf-β+IL4 vs WT CTRL, WT TGf-β+IL4 vs WT IL-4 and WT TGf-β+IL4 vs WT TGF-β.
