# Supplementary material for: Opposing functions of circadian protein DBP and atypical E2F family E2F8 in anti-tumor Th9 cell differentiation
Source: Nat Commun. 2022 Oct 14;13:6069. doi: 10.1038/s41467-022-33733-8 (PMC9568563; doi:10.1038/s41467-022-33733-8)
Supplement: Supplementary file 6 — Reporting Summary [file 41467_2022_33733_MOESM6_ESM.pdf]

nature portfolio

Corresponding author(s): Wanjun Chen

Last updated by author(s): Aug 17, 2022

Reporting Summary

Nature Portfolio wishes to improve the reproducibility of the work that we publish. This form provides structure for consistency and transparency in reporting. For further information on Nature Portfolio policies, see our Editorial Policies and the Editorial Policy Checklist.

Statistics

For all statistical analyses, confirm that the following items are present in the figure legend, table legend, main text, or Methods section.

n/a	Confirmed	
☐	☒	The exact sample size (*n*) for each experimental group/condition, given as a discrete number and unit of measurement
☐	☒	A statement on whether measurements were taken from distinct samples or whether the same sample was measured repeatedly
☐	☒	The statistical test(s) used AND whether they are one- or two-sided<br>*Only common tests should be described solely by name; describe more complex techniques in the Methods section.*
☒	☐	A description of all covariates tested
☐	☒	A description of any assumptions or corrections, such as tests of normality and adjustment for multiple comparisons
☐	☒	A full description of the statistical parameters including central tendency (e.g. means) or other basic estimates (e.g. regression coefficient) AND variation (e.g. standard deviation) or associated estimates of uncertainty (e.g. confidence intervals)
☐	☒	For null hypothesis testing, the test statistic (e.g. *F*, *t*, *r*) with confidence intervals, effect sizes, degrees of freedom and *P* value noted<br>*Give P values as exact values whenever suitable.*
☒	☐	For Bayesian analysis, information on the choice of priors and Markov chain Monte Carlo settings
☒	☐	For hierarchical and complex designs, identification of the appropriate level for tests and full reporting of outcomes
☒	☐	Estimates of effect sizes (e.g. Cohen's *d*, Pearson's *r*), indicating how they were calculated

Our web collection on statistics for biologists contains articles on many of the points above.

Software and code

Policy information about availability of computer code

Data collection	QuantStudio 3 Real-Time PCR Systems (Applied Biosystems), FACS Fortessa (BD Bioscience), Amersham Imager 600 (Cytiva)
Data analysis	BD FACSDiva 8.0.1 (Biosciences), FlowJo software v10.7.1 (TreeStar), GraphPad software Prism 8 (Graphpad), ImageJ v.1.53 (NIH)

For manuscripts utilizing custom algorithms or software that are central to the research but not yet described in published literature, software must be made available to editors and reviewers. We strongly encourage code deposition in a community repository (e.g. GitHub). See the Nature Portfolio guidelines for submitting code & software for further information.

Data

Policy information about availability of data

All manuscripts must include a data availability statement. This statement should provide the following information, where applicable:

- Accession codes, unique identifiers, or web links for publicly available datasets
- A description of any restrictions on data availability
- For clinical datasets or third party data, please ensure that the statement adheres to our policy

All array data support the findings of this study have been deposited in the NCBI Gene Expression Omnibus (GEO) database with the series accession number GSE182472. The authors declare that all other data supporting the findings of this study are available within the article and its supplementary information files. Source data are provided with this paper.

March 2021

Human research participants

Policy information about studies involving human research participants and Sex and Gender in Research.

Reporting on sex and gender	*Use the terms sex (biological attribute) and gender (shaped by social and cultural circumstances) carefully in order to avoid confusing both terms. Indicate if findings apply to only one sex or gender; describe whether sex and gender were considered in study design whether sex and/or gender was determined based on self-reporting or assigned and methods used. Provide in the source data disaggregated sex and gender data where this information has been collected, and consent has been obtained for sharing of individual-level data; provide overall numbers in this Reporting Summary. Please state if this information has not been collected. Report sex- and gender-based analyses where performed, justify reasons for lack of sex- and gender-based analysis.*
Population characteristics	*Describe the covariate-relevant population characteristics of the human research participants (e.g. age, genotypic information, past and current diagnosis and treatment categories). If you filled out the behavioural & social sciences study design questions and have nothing to add here, write "See above."*
Recruitment	*Describe how participants were recruited. Outline any potential self-selection bias or other biases that may be present and how these are likely to impact results.*
Ethics oversight	*Identify the organization(s) that approved the study protocol.*

Note that full information on the approval of the study protocol must also be provided in the manuscript.

Field-specific reporting

Please select the one below that is the best fit for your research. If you are not sure, read the appropriate sections before making your selection.

☒ Life sciences ☐ Behavioural & social sciences ☐ Ecological, evolutionary & environmental sciences

For a reference copy of the document with all sections, see nature.com/documents/nr-reporting-summary-flat.pdf

Life sciences study design

All studies must disclose on these points even when the disclosure is negative.

Sample size	Sample numbers were predetermined based on pilot studies and sample sizes were similar to generally employed in the field. We used sample sizes containing 3 or more biological replicates which can provide adequate statistical power in biological analysis.
Data exclusions	No data were excluded.
Replication	All experiments were replicated at least two times. And these replicated experiments were reliably reproduced.
Randomization	We did not use any randomization. Gender and age in each group were matched between control and experimental group.
Blinding	We did not perform blinding test, because this study was an observational study. The samples and animals were selected randomly and they ere gender and age matched.

Reporting for specific materials, systems and methods

We require information from authors about some types of materials, experimental systems and methods used in many studies. Here, indicate whether each material, system or method listed is relevant to your study. If you are not sure if a list item applies to your research, read the appropriate section before selecting a response.

Materials & experimental systems

n/a	Involved in the study	
☐	☒	Antibodies
☐	☒	Eukaryotic cell lines
☒	☐	Palaeontology and archaeology
☐	☒	Animals and other organisms
☒	☐	Clinical data
☒	☐	Dual use research of concern

Methods

n/a	Involved in the study	
☒	☐	ChIP-seq
☐	☒	Flow cytometry
☒	☐	MRI-based neuroimaging

Antibodies

Antibodies used	The following primary antibodies were used for flow cytometry; they are listed as antigen first, followed by supplier, fluorophore and catalog number as applicable.<br>anti-mouse CD3 (17A2) AF780 APC, eBioscience, # 47-0032-82<br>anti-mouse CD3 (OKT3) APC-eFluor450, eBioscience, # 48-0037-42<br>anti-mouse CD4 (RM4-5) PerCP, eBioscience, # 45-0042-82<br>anti-human CD4 (RPA-T4) FITC, eBioscience, # 11-0049-42<br>anti-mouse CD8a (53-6.7) FITC, eBioscience, # 11-0081-82<br>anti-mouse IL-10 (JES5-16E3) FITC, eBioscience, #17-7101-82<br>anti-mouse IL-13 (eBio13A) PE, eBioscience, #12-7133-82<br>anti-mouse IL-17 (eBio17B7) PE/Cyanine7, eBioscience, #25-7177-82<br>anti-mouse IFN-γ (XMG1.2) eFluor450, eBioscience, #48-7311-82<br>anti-mouse IL-4 (11B11) PE, eBioscience, #12-7041-82<br>anti-mouse Foxp3 (FJK-16s) Pacific blue, eBioscience, #48-5773-82<br>anti-mouse Granzyme B (NGZB) APC, eBioscience, #17-8898-82<br>anti-mouse IL-9 (RM9A4) APC, BioLegend, #514106<br>anti-human IL-9 (MH9A4) PE, BioLegend, #507605<br>anti-mouse TNF (MP6-XT22) FITC, BioLegend, #506304<br>anti-mouse IL-4 (11B11) PE/Cyanine7, BioLegend, #504118<br><br>The following primary antibodies were used for western blotting; they are listed as antigen first, followed by supplier, and catalog number as applicable.<br>phosphor-Smad3L-Thr179, Abcam, ab74062<br>phosphor-Smad3L-Ser204, Abcam, ab63402<br>phosphor-Smad3-Ser208, Abcam, ab138659<br>phosphor-Smad3L-Ser213, Abcam, ab63403<br>phosphor-Smad3 C-ter, Abcam, ab52903<br>Smad3, Abcam, ab75512<br>Dbp, Abcam, ab227591<br>E2f8, Abcam, ab109596<br>Phosphor-JNK, Cell Signaling Technology, 4668<br>total-JNK, Cell Signaling Technology, 9252<br>phosphor-ERK1/2, Cell Signaling Technology, 9101<br>total-ERK, Cell Signaling Technology, 9102<br>phosphor-p38, Cell Signaling Technology, 9211<br>total-p38, Cell Signaling Technology, 9212<br>phosphor-Stat5, Cell Signaling Technology, 9351<br>total-Stat5, Cell Signaling Technology, 94205<br>phosphor-Stat6, Santa Cruz, sc-11762<br>total-Stat6, Cell Signaling Technology, 9362<br>GAPDH, Cell Signaling Technology, 5174<br>horseradish peroxidase-conjugated anti-rabbit IgG, Cell Signaling Technology, 7074<br>horseradish peroxidase-conjugated anti-mouse IgG, Cell Signaling Technology, 7076
Validation	All antibodies were purchased from commercial companies (eBioscience, BioLegend, Invitrogen, Abcam, Cell Signaling), and validated by the manufacturers for identification of antigens and flow cytometry, western blot applications, as described on the manufacturers' websites.

Eukaryotic cell lines

Policy information about cell lines and Sex and Gender in Research

Cell line source(s)	We obtained HEK293T and B16F10 cell line from American Type Culture Collection (ATCC), and MCA205 from Merck.
Authentication	Cells have been authenticated in many experiments by transcript analysis and genome sequencing.
Mycoplasma contamination	The cell line was not tested for mycoplasma contamination.
Commonly misidentified lines (See ICLAC register)	No commonly misidentified cell lines were used.

Animals and other research organisms

Policy information about studies involving animals; ARRIVE guidelines recommended for reporting animal research, and Sex and Gender in Research

Laboratory animals	C57BL/6J wild-type, Smad3-/-, Tgfbr1f/f Cd4-cre+, Tgfbr1f/+ Cd4-cre−, Tgfbr1f/f ER-cre+, and Rag1-/- mice were used. Both sexes in

	the age of 8-10 weeks were used for experiments. All mice were fed free access to water and housed in a 12 h light/dark cycle-, temperature (23±3°C)- and humidity (range 40–60)-controlled room.
Wild animals	This study did not involve wild animals.
Reporting on sex	This information has not been collected.
Field-collected samples	No field-collected samples were used in this study.
Ethics oversight	All animal studies were performed according to US National Institutes of Health guidelines for the use and care of live animals and approved by the Animal Care and Use Committees of National Institute of Dental and Craniofacial Research.

Note that full information on the approval of the study protocol must also be provided in the manuscript.

Flow Cytometry

Plots

Confirm that:

- [x] The axis labels state the marker and fluorochrome used (e.g. CD4-FITC).
- [x] The axis scales are clearly visible. Include numbers along axes only for bottom left plot of group (a 'group' is an analysis of identical markers).
- [x] All plots are contour plots with outliers or pseudocolor plots.
- [x] A numerical value for number of cells or percentage (with statistics) is provided.

Methodology

Sample preparation	The cells were stained with antibodies specific to the various surface molecules, fixed and permeabilized with Fixation/ Permeabilization buffer solution according to the manufacturer's protocol (eBioscience).
Instrument	BD LSRII Fortessa
Software	BD FACSDiva 8.0.1 (Biosciences), FlowJo 10.7.1 (Tree Star)
Cell population abundance	The abundance of the relevant cell population was 20,000 cells per case.
Gating strategy	All FACS analysis was performed in T cells. CD4+ T cells were gated as: single (FSC-W/FSC-H), live (Zombie yellow-), CD4 T (CD4+). The boundaries between positive and negative populations (IL-9) were defined based on isotype staining. For analyzing different cells, cells were stained with specific antibodies.

- [x] Tick this box to confirm that a figure exemplifying the gating strategy is provided in the Supplementary Information.
